# *Bifidobacterium longum* B18 Modulates Pharmacologically Induced Sleep Parameters in Rats in Association with Altered Tryptophan–Melatonin Metabolism

**DOI:** 10.3390/foods15183230

**Published:** 2026-09-12

**Authors:** Lili Liang, Qiaoxuan Ke, Lanyan Huang, Shaofang Hu, Weipeng Guo, Weiyi Luo, Ling Chen, Qingping Wu

**Affiliations:** 1 College of Food Science, South China Agricultural University, Guangzhou 510642, China; lililiang9302@126.com; 2 State Key Laboratory of Applied Microbiology Southern China, Guangdong Provincial Key Laboratory of Microbial Safety and Health, National Health Commission Science and Technology Innovation Platform for Nutrition and Safety of Microbial Food, Key Laboratory of Big Data Technologies for Food Microbiological Safety, State Administration for Market Regulation, Institute of Microbiology, Guangdong Academy of Sciences, Guangzhou 510070, China; 3 Food and Drug Laboratory, Guangdong Detection Centre of Microbiology, Guangzhou 510070, China

**Keywords:** *Bifidobacterium longum* B18, sleep, gut microbiota, tryptophan metabolism, melatonin, functional foods

## Abstract

Sleep disorders represent a growing public health concern, yet current pharmacological interventions are limited by tolerance, dependence, and rebound insomnia. This study evaluated the probiotic properties of *Bifidobacterium longum* B18 and its effects on pharmacologically induced sleep parameters in male Sprague–Dawley rats, focusing on tryptophan-related metabolism and gut microbiota composition. In vitro, B18 exhibited favourable probiotic characteristics, no detectable haemolytic activity, and susceptibility to all eight antimicrobials tested. In vivo, B18 significantly shortened barbital sodium-induced sleep latency and prolonged pentobarbital sodium-induced sleep duration (*p* < 0.05). Targeted metabolomic profiling of faeces, plasma, and hippocampus showed increased tryptophan and melatonin concentrations, whereas 5-HTP and 5-HT remained unchanged. B18 was also associated with lower Kyn/Trp ratios, although plasma Kyn concentrations were unchanged. Increased MT/5-HT ratios and elevated hippocampal AANAT levels were also observed, supporting an association with enhanced melatonin-related metabolism. Gut microbiota analysis showed higher abundances of *Bifidobacterium* and *Lactobacillus* in the B18 group than in the DZP group, together with lower *Desulfovibrio* abundance than in the CTR. Exploratory correlation analysis further showed positive associations of *Bifidobacterium* abundance with faecal tryptophan and melatonin levels and an inverse association with the Kyn/Trp ratio. Collectively, these findings indicate that *B. longum* B18 modifies pharmacologically induced sleep parameters in association with altered tryptophan–melatonin metabolism and gut microbiota composition, supporting further evaluation of B18 as a candidate probiotic for sleep-related functional applications.

## 1. Introduction

Sleep, which occupies approximately one-third of the human lifespan, plays indispensable roles in neural homeostasis and synaptic plasticity [1], metabolic homeostasis [2,3], and immune regulation [4], and its disruption has been linked to impaired cognition [5,6], emotional dysregulation [7,8,9], and metabolic disorders [10,11]. Insomnia, the most prevalent sleep disorder, affects an estimated 10–30% of the global population and is characterised by difficulty initiating and maintaining sleep and by premature awakening [12,13]. Current pharmacological management, including benzodiazepine receptor agonists [14], melatonin-based preparations [15], and antihistaminergic agents [16], is limited by tolerance, dependence risk, and rebound sleep disturbance upon withdrawal. These limitations have driven growing interest in non-pharmacological approaches, particularly microbiota-targeted strategies that modulate the gut–brain axis, underscoring the scientific and practical importance of developing safe and effective functional foods and dietary interventions to improve sleep.

As a critical nexus linking gut microbiota to central nervous system function, the gut–brain axis mediates bidirectional neuroendocrine–immune crosstalk, with tryptophan (Trp) serving as a central metabolic hub in this signalling network. Neurochemical homeostasis, circadian rhythmicity, and sleep architecture may be influenced by the relative allocation of Trp among three major metabolic branches: serotonin/melatonin biosynthesis, kynurenine pathway metabolism, and indole derivative production, each of which can be modulated by gut microbiota composition [17,18]. Under physiological conditions, the kynurenine pathway represents the predominant route of Trp catabolism, accounting for approximately 90% of systemic Trp degradation. Within this pathway, indoleamine 2,3-dioxygenase 1 (IDO1)-mediated conversion of Trp to Kyn is an important regulatory step [19], particularly under immune- or inflammation-related conditions. By contrast, only a small proportion of Trp is directed toward melatonin (MT) biosynthesis via the serotonergic pathway, with arylalkylamine N-acetyltransferase (AANAT) catalysing the rate-limiting step. Gut microbiota composition has been reported to influence tryptophan metabolism. In other experimental contexts, specific *Bifidobacterium* [20] and *Lactobacillus* [21] strains have been associated with reduced IDO-related kynurenine pathway activity, suggesting a potential role in preserving Trp availability. In contrast, potentially pathogenic taxa such as *Desulfovibrio* may contribute to a pro-inflammatory milieu characterised by enhanced LPS-driven cytokine signalling [22]. Inflammatory conditions have been proposed to modulate IDO1-related tryptophan metabolism and kynurenine pathway activity [23,24]; however, direct mechanistic evidence linking inflammation-induced IDO1 upregulation to altered Trp flux remains limited and warrants further investigation.

Within the evolving framework of the microbiota–gut–brain axis, psychobiotics—probiotics that confer neurological benefits through microbiota-mediated gut–brain communication—have attracted considerable research interest, with several *Lactobacillus* and *Bifidobacterium* strains reported to improve sleep-related outcomes through mechanisms involving neurotransmitter metabolism, gut microbiota remodelling, and neuroimmune signalling [25,26,27,28]. However, the pronounced strain-specificity of these effects, together with the limited understanding of how probiotic interventions influence kynurenine metabolism and AANAT-associated melatonin biosynthesis, underscores the need to systematically characterise novel candidate strains and their underlying metabolic effects.

*Bifidobacterium longum* B18 is a novel strain whose probiotic properties and potential effects on sleep parameters have not previously been characterised. We hypothesised that B18 supplementation may improve sleep parameters and be associated with alterations in gut microbiota composition and Trp metabolism. This study therefore sought to: (i) characterise the in vitro safety and probiotic properties of B18; (ii) assess its effects on sleep-related outcomes in a pharmacologically induced rat model; (iii) perform targeted profiling of Trp-related metabolites across faecal, plasma, and hippocampal compartments; and (iv) characterise changes in gut microbiota composition and explore their associations with Trp metabolism and sleep parameters. Collectively, this study aimed to provide insight into the potential involvement of the microbiota–gut–brain axis in the effects of B18 on sleep parameters.

## 2. Materials and Methods

### 2.1. Antimicrobial Susceptibility of Bifidobacterium longum B18

Antimicrobial susceptibility of *Bifidobacterium longum* B18 to ampicillin, vancomycin, gentamicin, streptomycin, erythromycin, clindamycin, tetracycline, and chloramphenicol (all purchased from Shanghai Yuanye Bio-Technology Co., Ltd., Shanghai, China) was determined by broth microdilution in accordance with EFSA guidance [29]. Twofold serial dilutions of each antimicrobial were prepared, and B18 was incubated anaerobically (85% N_2_, 10% H_2_, and 5% CO_2_) at 37 °C for 48 h. The MIC was defined as the lowest antimicrobial concentration that completely inhibited visible growth. MIC values were interpreted using the EFSA microbiological cut-off values for *Bifidobacterium*. MIC values at or below the corresponding microbiological cut-off were interpreted as indicating phenotypic susceptibility, whereas values above the cut-off were interpreted as indicating phenotypic resistance and requiring further evaluation of the potential genetic basis of resistance.

### 2.2. Haemolytic Activity of Bifidobacterium longum B18

We conducted the hemolysis experiment as described by Zhang et al. to evaluate whether B18 exhibits haemolytic activity [30]. A 5 µL aliquot of the cultured B18 suspension was inoculated onto laboratory-prepared mMRS agar plates supplemented with 5% (*v*/*v*) sheep blood. The plates were incubated anaerobically in a Don Whitley Scientific anaerobic workstation (Don Whitley Scientific Limited, Bingley, UK) under an atmosphere of 85% N_2_, 10% H_2_, and 5% CO_2_ at 37 °C for 36 h. *Staphylococcus aureus* ATCC 25923 was used as a β-haemolytic positive control. β-haemolysis was identified by the formation of a clear zone surrounding the colonies, α-haemolysis by greenish or brownish discoloration of the surrounding medium, and γ-haemolysis by the absence of visible hemolysis or discoloration around the colonies.

### 2.3. B. longum B18 Culture and Preparation

Stock cultures of *B. longum* B18, originally isolated from faecal samples of a healthy 3-year-old child in Xinyang, Henan, China, are preserved in the Guangdong Microbial Culture Collection centre (accession no. M2CF22M7). Prior to each experiment, the strain was revived through two successive subcultures on modified de Man, Rogosa and Sharpe (mMRS) agar in a Don Whitley Scientific anaerobic workstation under an atmosphere of 85% N_2_, 10% H_2_, and 5% CO_2_ at 37 °C for 24–36 h. The revived culture was subsequently inoculated (2%, *v*/*v*) into mMRS broth, which was prepared according to the formulation provided in Supplementary Table S13 without the addition of agar, and incubated under the same anaerobic conditions at 37 °C for 24–36 h. 

For the animal experiments, B18 cells in the late logarithmic phase were harvested by centrifugation (5000× *g*, 10 min, 4 °C), washed twice with PBS, and resuspended in sterile physiological saline to a final concentration of approximately 1 × 10^9^ CFU/mL for intragastric administration. Details regarding the standardisation of the B18 suspension during the 35-day intervention and the rationale for dose selection are provided in Supplementary Section S1.13.

### 2.4. Tolerance of B. longum B18 to Simulated Gastric and Intestinal Fluids

Tolerance to simulated gastrointestinal conditions was evaluated using an in vitro assay adapted from Wang et al. [31]. *B. longum* B18 was cultured and harvested as described in Section 2.3 and resuspended to a final density of 1 × 10^8^ CFU/mL. Under the same anaerobic atmosphere described in Section 2.3. The bacterial suspension (1 mL) was added to 9 mL of simulated gastric fluid (pH 3.0) containing pepsin (Shanghai Yuanye Bio-Technology Co., Ltd., Shanghai, China) at a final concentration of 3 mg/mL and incubated anaerobically at 37 °C, with viable counts determined at 0 and 3 h. Subsequently, 1 mL of the gastric-treated suspension was transferred into 9 mL of simulated intestinal fluid (pH 8.0) containing trypsin (Shanghai Yuanye Bio-Technology Co., Ltd., Shanghai, China) at a final concentration of 1 mg/mL and bovine bile salts (Shanghai Yuanye Bio-Technology Co., Ltd., Shanghai, China) at 0.3% (*w*/*v*), followed by anaerobic incubation at 37 °C for a further 3 h. Viable counts were then determined. All assays were performed in triplicate, and the survival rate of B18 was calculated as follows:

$$K_{1}\;=\frac{N_{t}}{\;N_{0}}\times 100\%$$

where *K*_1_ is the survival rate of B18 (%), *N_t_* is the viable count of *B. longum* B18 after 3 h of exposure to simulated gastric or intestinal fluid (CFU), and *N*_0_ is the viable count of *B. longum* B18 at 0 h in simulated gastric or intestinal fluid (CFU).

### 2.5. Determination of Auto-Aggregation of B. longum B18

Auto-aggregation ability was evaluated using a modified version of a previously reported method [32]. *B. longum* B18 was cultured and harvested as described in Section 2.3 and resuspended in 3 mL of physiological saline. Under the same anaerobic atmosphere described in Section 2.3. An aliquot (200 μL) was immediately withdrawn for absorbance measurement at 600 nm (designated as *K*_0_), after which the remaining suspension was left undisturbed at room temperature; absorbance readings were taken at 3, 6, and 9 h (designated as *K_t_*). All assays were performed in triplicate, and the auto-aggregation rate was calculated as follows:

$$K_{2}\;=\;(1\;-\;\frac{K_{t}}{K_{0}})\;\times 100\%$$

where *K*_2_ represents the auto-aggregation ability of *B. longum* B18 (%), *K***_0_** is the absorbance at 600 nm at 0 h, and *K**_t_*** is the absorbance at 600 nm after standing for different times (t = 3, 6, and 9 h).

### 2.6. Co-Aggregation of B. longum B18 with Enterohemorrhagic Escherichia coli O157:H7

The Co-aggregation ability of *B. longum* B18 with enterohemorrhagic *Escherichia coli* O157:H7 was evaluated using a modified version of a previously reported method [33]. *B. longum* B18 was cultured and harvested as described in Section 2.3 and resuspended in physiological saline. Under the same anaerobic atmosphere described in Section 2.3. Equal volumes of the B18 suspension and the *E. coli* O157:H7 suspension were combined, vortexed for 10 s, and incubated at 37 °C. Aliquots (200 μL) of the upper suspension were withdrawn at 3, 6, and 9 h for absorbance measurement at 600 nm, with all assays performed in triplicate. The co-aggregation rate of B18 was calculated as follows:

$$K_{3}\;=\;\frac{1-2\times B_{mix}}{B_{p}+B_{E}}\times 100\%$$

where *K*_3_ represents the co-aggregation ability of *B. longum* B18 (%), *B_P_* is the initial absorbance of *B. longum* B18, *B_E_* is the initial absorbance of *E. coli* O157:H7, and *B_mix_* is the absorbance of the mixed suspension of *B. longum* B18 and *E. coli* O157:H7 after incubation for 3, 6, or 9 h.

### 2.7. Animal Experimental Design

Specific pathogen-free (SPF) male Sprague–Dawley rats (8 weeks old) were purchased from Zhuhai Baishitong Biotechnology Co., Ltd. (Zhuhai, China).. All animals were housed in a barrier facility under controlled environmental conditions at 22 ± 3 °C with a 12 h light/12 h dark cycle, and were allowed free access to standard chow and autoclaved water. After one week of acclimatization, the rats were randomly assigned to different groups for subsequent experiments. All experimental procedures involving animals were approved by the Institutional Animal Care and Use Committee of the Institute of Microbiology, Guangdong Academy of Sciences (Approval No. GT-IACUC202308041). Detailed criteria for sleep assessment, randomisation and blinding procedures, and the determination of suprathreshold and subthreshold drug doses are provided in Supplementary Sections S1.2–S1.6.

### 2.8. Evaluation of the Effects of B. longum B18 on Pentobarbital Sodium-Induced Sleep Duration and Barbital Sodium-Induced Sleep Latency in Rats

A total of 30 rats were randomly divided into three groups (*n* = 10 per group). The normal control group (CTR) received saline (1 mL/rat) by intragastric administration, the probiotic group (B18) received a *B. longum* B18 suspension (1 × 10^9^ CFU/mL, 1 mL/rat), and the positive control group (DZP) received diazepam (Jining Ankang Pharmaceutical Co., Ltd., Jining, China) at a dose equivalent to 3 mg/kg diazepam (1 mL/rat) for 35 consecutive days. On day 30, 30 min after gavage, all rats were intraperitoneally injected with pentobarbital sodium (50 mg/kg, Sigma-Aldrich, St. Louis, MO, USA) at the minimum supra-threshold dose, and sleep duration was recorded. On day 35, 30 min after gavage, all rats were intraperitoneally injected with barbital sodium (300 mg/kg, Sigma-Aldrich, St. Louis, MO, USA) at the minimum supra-threshold dose, and sleep latency was recorded. Sleep duration was defined as the interval between the loss and recovery of the righting reflex following pentobarbital sodium administration, whereas sleep latency was defined as the interval between barbital sodium administration and the loss of the righting reflex. Detailed criteria for sleep onset, awakening, sleep latency, and sleep duration are provided in Supplementary Section S1.2.

### 2.9. Timing of Experimental Procedures

Rats were maintained under a 12 h light/12 h dark cycle, with lights on at 08:00 (defined as ZT0) and lights off at 20:00 (ZT12). B18 was administered by oral gavage at approximately 10:00 (ZT2). Pharmacological sleep tests were conducted between 11:00 and 13:00 (ZT3–ZT5). Following the assessments, the rats were monitored until recovery of the righting reflex before being returned to their home cages. Terminal sample collection was performed 24 h after completion of the barbital sodium-induced sleep test. Blood, hippocampal tissue, and colonic faecal contents were collected between 13:00 and 15:00 (ZT5–ZT7). The sampling order was alternated among the experimental groups to minimise potential temporal bias.

### 2.10. UHPLC–MS/MS Analysis of Tryptophan-Related Metabolites

Tryptophan and its downstream metabolites—Trp, 5-hydroxytryptophan (5-HTP), 5-hydroxytryptamine (5-HT), melatonin (MT), kynurenine (Kyn), and 5-hydroxyindoleacetic acid (5-HIAA)—were quantified in plasma (100 μL), faecal samples (60 mg) and hippocampal tissue (60 mg) by UHPLC–MS/MS (Thermo Fisher Scientific, Germering, Germany). Analytical standards of these metabolites were purchased from Shanghai Yuanye Bio-Technology Co., Ltd. (Shanghai, China). Faecal and hippocampal samples were homogenised prior to extraction. All samples were deproteinized with pre-chilled methanol containing [^15^N_2_]-L-tryptophan and indole-3-acetic acid-d_7_ (both from Sigma-Aldrich, St. Louis, MO, USA) and melatonin-d_4_ (TMstandard, Changzhou, China). The samples were then held at −20 °C for 2 h. Following centrifugation (20,000 rpm, 15 min, 4 °C), supernatants were dried under nitrogen and reconstituted in 100 μL methanol (0.1% formic acid) prior to injection (3 μL) onto a SB-C18 RRHD column (1.8 um, 2.1 × 100 mm; Agilent Technologies, Santa Clara, CA, USA). Chromatographic separation was achieved using water (0.2% formic acid; A) and methanol (0.2% formic acid; B) as mobile phases at 0.3 mL/min, with the following gradient: 0–1 min, 1% B; 1–5 min, 1–50% B; 5–8 min, 50–95% B; 8–10 min, 95% B; 10–10.5 min, 95–1% B. 

### 2.11. 16S rRNA Gene Sequencing and Gut Microbiota Analysis

Faecal pellets were collected from each rat on the final day of the intervention, flash-frozen in liquid nitrogen, and stored at −80 °C until DNA extraction. For microbiota analysis, 200 mg of faecal material from each rat was used for genomic DNA extraction with a commercial kit (Qiagen, Hilden, Germany) according to the manufacturer’s instructions. The V3–V4 region of the bacterial 16S rRNA gene was amplified with primers 338F/806R and sequenced on an Illumina MiSeq platform. Alpha and beta diversity (binary Euclidean, PCoA) were calculated, and differentially abundant taxa were identified by LEfSe (LDA score > 2). The Bacillota/Bacteroidota ratio was derived from phylum-level relative abundances.

### 2.12. Animal Allocation and Sample Selection

All 10 rats in each group were included in the behavioural assessments. Six rats per group were randomly selected from the original cohort for biochemical and metabolomic analyses. Of these six animals, five were further randomly selected for 16S rRNA gene sequencing, thereby allowing microbiota and metabolite data to be matched at the individual-animal level. Sample selection was independent of the experimental outcomes, and no animals or samples were excluded because of insufficient sample volume, DNA quality, analytical failure, or unexpected experimental results. Exploratory Spearman correlation analyses were performed between differentially abundant bacterial genera and faecal tryptophan-related metabolites and metabolic ratios using individually matched microbiota and metabolite data from 15 animals (n = 5 per group; n = 15 in total). *p*-values were adjusted for multiple comparisons using the Benjamini–Hochberg false discovery rate (FDR) procedure, and q < 0.05 was considered statistically significant.

### 2.13. Determination of Hippocampal AANAT Levels

Hippocampal tissues were homogenised on ice in pre-cooled PBS (0.01 M, pH 7.4), and the homogenates were centrifuged at 5000× *g* for 10 min. The resulting supernatants were collected for AANAT determination. Hippocampal AANAT concentrations were quantified using a Rat Arylalkylamine-N-acetyltransferase (AANAT) ELISA kit (catalogue no. DG20818D, Dogesce, Beijing, China) according to the manufacturer’s instructions. The assay was based on a sandwich ELISA using an AANAT-specific capture antibody and an HRP-conjugated detection antibody. Briefly, 50 μL of each sample or standard was added to the appropriate well, followed by 100 μL of HRP-conjugated detection antibody and incubation at 37 °C for 60 min. After washing, substrate solutions were added and incubated at 37 °C for 15 min in the dark. The reaction was stopped with stop solution, and absorbance was measured at 450 nm. AANAT concentrations were calculated from the standard curve. Total protein concentrations of the corresponding hippocampal homogenates were measured separately, and AANAT levels were normalised to total protein content and expressed as ng AANAT per mg total protein (ng/mg protein).

## 3. Statistical Analysis

All statistical analyses were conducted using GraphPad Prism 9.0 (GraphPad Software, San Diego, CA, USA), except for gut microbiome analyses. Data are presented as mean ± SD. Data normality was assessed using the Shapiro–Wilk test. Normally distributed data were analysed using Student’s *t*-test for comparisons between two groups or one-way analysis of variance (ANOVA) followed by Tukey’s post hoc test for multiple-group comparisons. Non-normally distributed data were analysed using the Kruskal–Wallis rank-sum test followed by Dunn’s multiple comparisons test for post hoc pairwise comparisons. Categorical variables were analysed using the chi-square (χ^2^) test. A *p*-value < 0.05 was considered statistically significant.

## 4. Results

### 4.1. Haemolytic Activity and Antibiotic Susceptibility of B. longum B18

**Figure 1:**
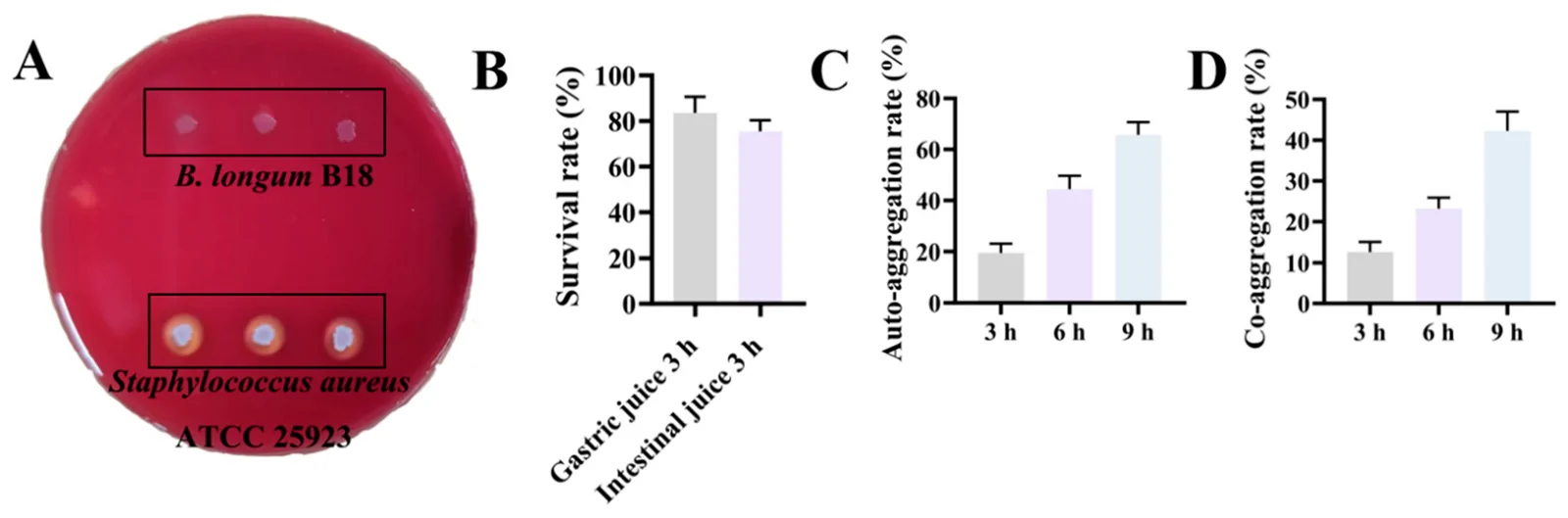
Haemolytic activity and probiotic properties of *B. longum* B18. (**A**) Haemolytic activity of B18 using *Staphylococcus aureus* ATCC 25923 as a positive control. Probiotic properties included, (**B**) tolerance to simulated gastric and intestinal fluids, (**C**) auto-aggregation ability, and (**D**) co-aggregation ability. Values are expressed as mean ± SD from three independent experiments performed using independently prepared B18 cultures (*n* = 3).

As shown in Figure 1A, growth of B18 on 5% sheep blood agar did not result in any visible haemolysis, illustrating that B18 was γ-haemolytic and did not lyse red blood cells. *B. longum* B18 was susceptible to all eight antimicrobials tested based on the EFSA microbiological cut-off values (Table 1). The MICs of ampicillin, vancomycin, gentamicin, streptomycin, erythromycin, clindamycin, tetracycline, and chloramphenicol were 1, 0.5, 16, 32, 0.5, 0.25, 2, and 2 μg mL^−1^, respectively. All MIC values were below the corresponding EFSA microbiological cut-off values, indicating that B18 exhibited a susceptible phenotype toward all antimicrobials examined. 

### 4.2. Tolerance of B. longum B18 to Simulated Gastrointestinal Fluids

As shown in Figure 1B, *B. longum* B18 exhibited a survival rate of 83.88 ± 4.56% after 3 h of treatment with simulated gastric fluid, which remained at 76.78 ± 3.20% after a subsequent 3 h exposure to simulated intestinal fluid. These findings suggest that B18 retains high viability under both gastric and intestinal challenge conditions and exhibits robust gastrointestinal tolerance.

### 4.3. B. longum B18 Exhibits Favorable Auto-Aggregation and Co-Aggregation Abilities

As shown in Figure 1C,D, the auto-aggregation rate of B18 increased progressively with incubation time and reached 65.73 ± 5.01% at 9 h, indicating a relatively high auto-aggregation capacity. The co-aggregation ability of B18 with *Escherichia coli* ranged from 12.52% to 42.29%, with the highest value observed after 9 h of co-incubation (42.29 ± 4.71%). These results indicate that B18 possesses good auto-aggregation and co-aggregation properties, which may support its adhesion and persistence in the host intestinal tract while contributing to the competitive exclusion of pathogenic bacteria.

### 4.4. Surface Hydrophobicity of B. longum B18

B18 exhibited relatively high surface hydrophobicity in the xylene system, with a hydrophobicity value of 62.86 ± 4.45% (Table 2, see Supplementary Section S1.1 for the detailed assay procedure), indicating that it is a strain with high surface hydrophobicity. These findings suggest that *B. longum* B18 possesses strong surface hydrophobic characteristics, which may contribute to its adhesion potential. 

### 4.5. B. longum B18 Prolongs Pentobarbital Sodium-Induced Sleep Duration and Reduces Barbital Sodium-Induced Sleep Latency in Rats

**Figure 2:**
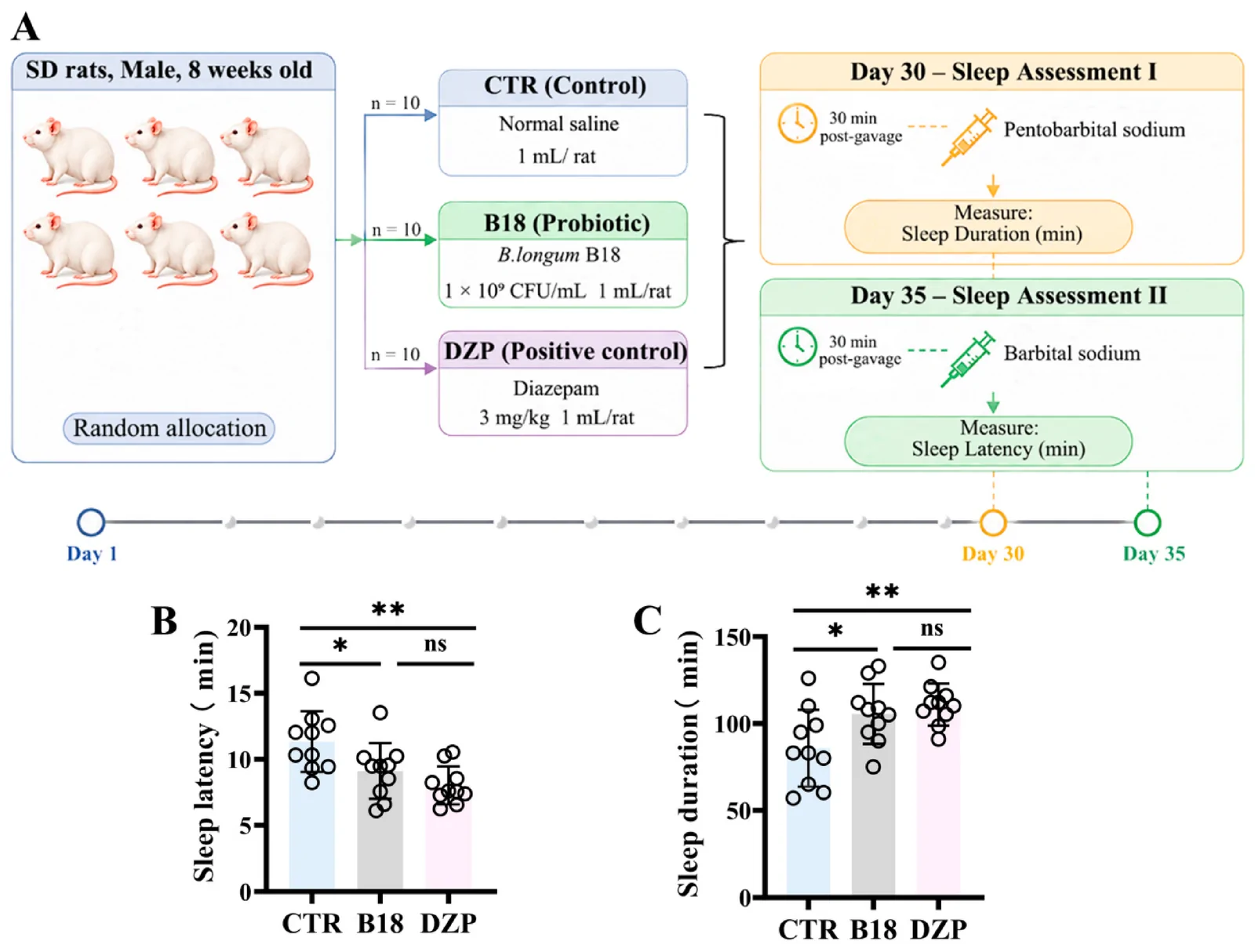
Experimental design and evaluation of the sleep-promoting activity of *B. longum* B18 in a pharmacologically induced rat sleep model. (**A**). Experimental design for evaluating the sleep-promoting effects of *B. longum* B18 in SD rats using pentobarbital sodium-induced sleep duration and barbital sodium-induced sleep latency assays. (**B**) Effects of *B. longum* B18 on barbital-induced sleep latency in rats. (**C**) Effects of *B. longum* B18 on pentobarbital sodium-induced sleep duration. *n* = 10 rats per group. Data are presented as mean ± SD. Statistical significance was determined by one-way ANOVA followed by Tukey’s post hoc test. ns, not significant; * *p* < 0.05; ** *p* < 0.01.

Thirty minutes after gavage, rats in each group received an intraperitoneal injection of the minimum suprathreshold doses of pentobarbital sodium (50 mg/kg) or barbital sodium (300 mg/kg) to evaluate the effects of *B. longum* B18 on sleep-related behaviours. Compared with the CTR, B18 treatment significantly shortened sleep latency in barbital sodium–treated rats and significantly prolonged sleep duration in pentobarbital sodium–treated rats (Figure 2B,C; *p* < 0.05), suggesting that B18 may enhance the hypnotic effects of both barbital sodium and pentobarbital sodium.

### 4.6. Effects of B. longum B18 on Tryptophan-Related Metabolites in the Plasma, Feces, and Hippocampus of Rats

**Figure 3:**
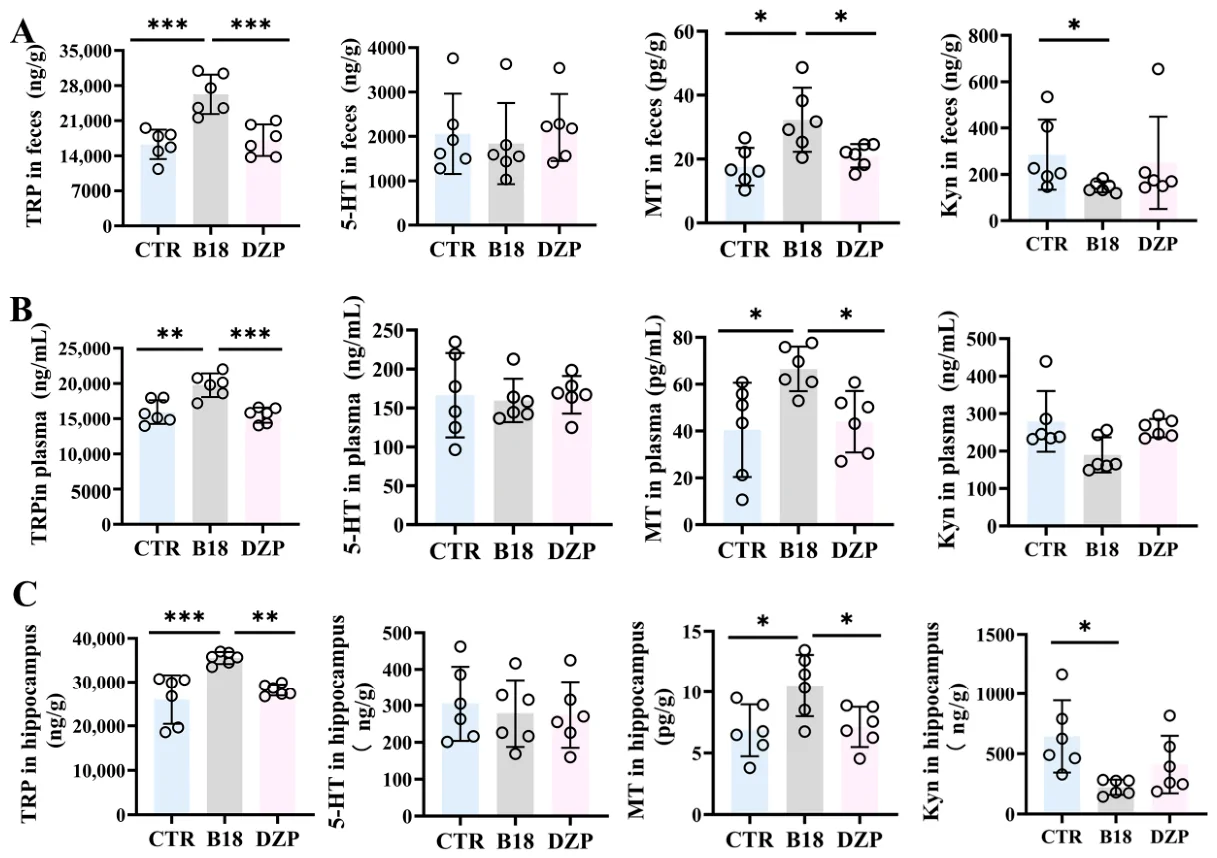
Effects of *B. longum* B18 on the levels of Trp, 5-HT, 5-HTP, MT, and Kyn in the feces, plasma, and hippocampus of rats. (**A**) Feces; (**B**) Plasma; (**C**) Hippocampus. *n* = 6 rats per group. Data are presented as mean ± SD. Statistical significance was determined by one-way ANOVA followed by Tukey’s post hoc test. * *p* < 0.05, ** *p* < 0.01, ***< 0.001.

Targeted metabolomic analysis of tryptophan-related metabolites showed that B18 supplementation significantly altered metabolic profiles in faeces, plasma and hippocampus. Trp levels were markedly higher in the B18 group than in both the CTR and DZP groups across all three sample types (Figure 3, *p* < 0.01). Similarly, MT levels in faeces, plasma and hippocampus were all significantly elevated following B18 treatment relative to the CTR and DZP groups (*p* < 0.05). However, faecal, plasma, and hippocampal concentrations of 5-HT, 5-HTP, and 5-HIAA did not differ significantly among groups (Figure 3 and Figure S1, *p* > 0.05). Faecal and hippocampal Kyn concentrations were markedly attenuated in B18-treated rats relative to the CTR (Figure 3A,C, *p* < 0.05), whereas plasma Kyn remained unchanged (Figure 3B, *p* > 0.05). Collectively, these findings indicate that B18 supplementation is associated with alterations in tryptophan-related metabolism.

### 4.7. B. longum B18 Modulates Pharmacologically Induced Sleep Parameters in Association with Altered Tryptophan Metabolism

**Figure 4:**
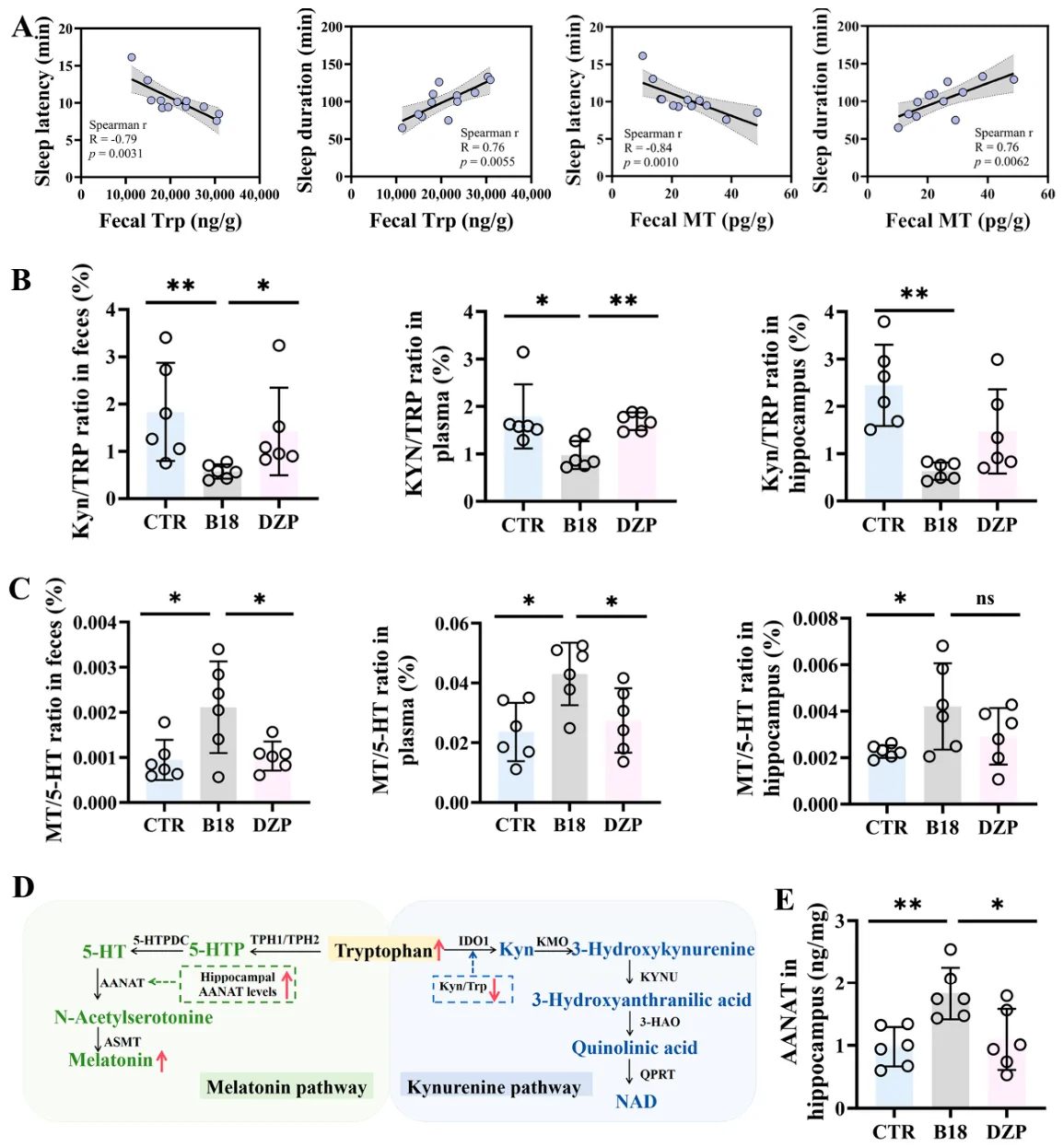
*B. longum* B18 is associated with altered tryptophan–melatonin metabolism and pharmacologically induced sleep parameters. (**A**) Spearman correlation analysis between faecal Trp and MT levels and sleep behavioural parameters. Correlations were calculated using paired data from the CTR and B18 groups (n = 6 per group). (**B**) Kyn/Trp ratios in faeces, plasma and hippocampus among the CTR, B18 and DZP groups. (**C**) MT/5-HT ratios in faeces, plasma and hippocampus among the CTR, B18 and DZP groups. (**D**) Schematic illustration of the proposed model by which *B. longum* B18 is associated with altered tryptophan metabolism, characterised by a reduced Kyn/Trp ratio and enhanced melatonin-related metabolism. (**E**) Hippocampal AANAT levels in the CTR, B18 and DZP groups. n = 6 rats per group. Data are presented as mean ± SD, with individual data points shown for each animal. Statistical significance is indicated as * *p* < 0.05 and ** *p* < 0.01; ns, not significant.

To examine the association between tryptophan metabolism and sleep parameters, Spearman rank correlations were computed between faecal Trp and MT levels and the two sleep-related parameters. As shown in Figure 4A, faecal Trp levels showed a significant inverse association with barbital sodium–induced sleep latency (*r* = −0.79, *p* = 0.0031) and a significant positive association with pentobarbital sodium–induced sleep duration (*r* = 0.76, *p* = 0.0055). Consistently, faecal MT levels exhibited a significant negative correlation with sleep latency (*r* = −0.84, *p* = 0.0010) and a significant positive correlation with sleep duration (*r* = 0.76, *p* = 0.0062). Collectively, these correlations indicate that higher faecal Trp and MT levels were associated with shorter barbital sodium-induced sleep latency and longer pentobarbital sodium-induced sleep duration. 

To assess the relative balance of Kyn and Trp after B18 intervention, Kyn/Trp ratios were calculated in faeces, plasma, and hippocampus (Figure 4B). The ratio was lower in the B18 group than in the CTR (*p* < 0.01) and DZP groups (*p* < 0.05) in both faeces and plasma. A lower ratio was also observed in the hippocampus compared with the CTR (*p* < 0.01). The metabolite changes underlying these ratios differed among matrices. In faeces and hippocampus, the lower ratios were accompanied by higher Trp and lower Kyn concentrations relative to the CTR. In plasma, Kyn remained unchanged, and the lower ratio reflected the increase in Trp. These results indicate that B18 supplementation altered the relative balance between Trp and Kyn.

To assess whether the elevated MT levels observed following B18 intervention were accompanied by changes in melatonin-related metabolism, the MT/5-HT ratio was calculated (Figure 4C). In faeces and plasma, the MT/5-HT ratio was markedly elevated in the B18 group relative to both CTR and DZP groups (*p* < 0.05). In the hippocampus, B18 treatment increased the MT/5-HT ratio relative to the CTR (*p* < 0.05), whereas values remained comparable to those observed in the DZP group (*p* > 0.05, ns). To further examine the potential involvement of AANAT in hippocampal melatonin-related metabolism, hippocampal AANAT levels were quantified (Figure 4E). Hippocampal AANAT levels were significantly higher in the B18 group than in both the CTR (*p* < 0.01) and DZP groups (*p* < 0.05). Together with the increased hippocampal MT concentration, these findings suggest that B18 supplementation may be associated with altered AANAT-related melatonin metabolism.

Based on these findings, Figure 4D presents an interpretive model summarising the associations between *B. longum* B18 supplementation and alterations in tryptophan-related metabolism. B18 supplementation was associated with reduced Kyn/Trp ratios across the three biological matrices, suggesting altered kynurenine-related tryptophan metabolism. Concurrently, increased hippocampal AANAT levels, together with elevated MT/5-HT ratios and MT concentrations, support the potential involvement of AANAT-related melatonin metabolism. These metabolic patterns were observed alongside shortened barbital sodium-induced sleep latency and prolonged pentobarbital sodium-induced sleep duration following B18 supplementation. The proposed model summarises these associations but does not establish causal relationships between the metabolic alterations and sleep-related outcomes.

### 4.8. Gut Microbiota Composition in Rats Following B. longum B18 Supplementation

**Figure 5:**
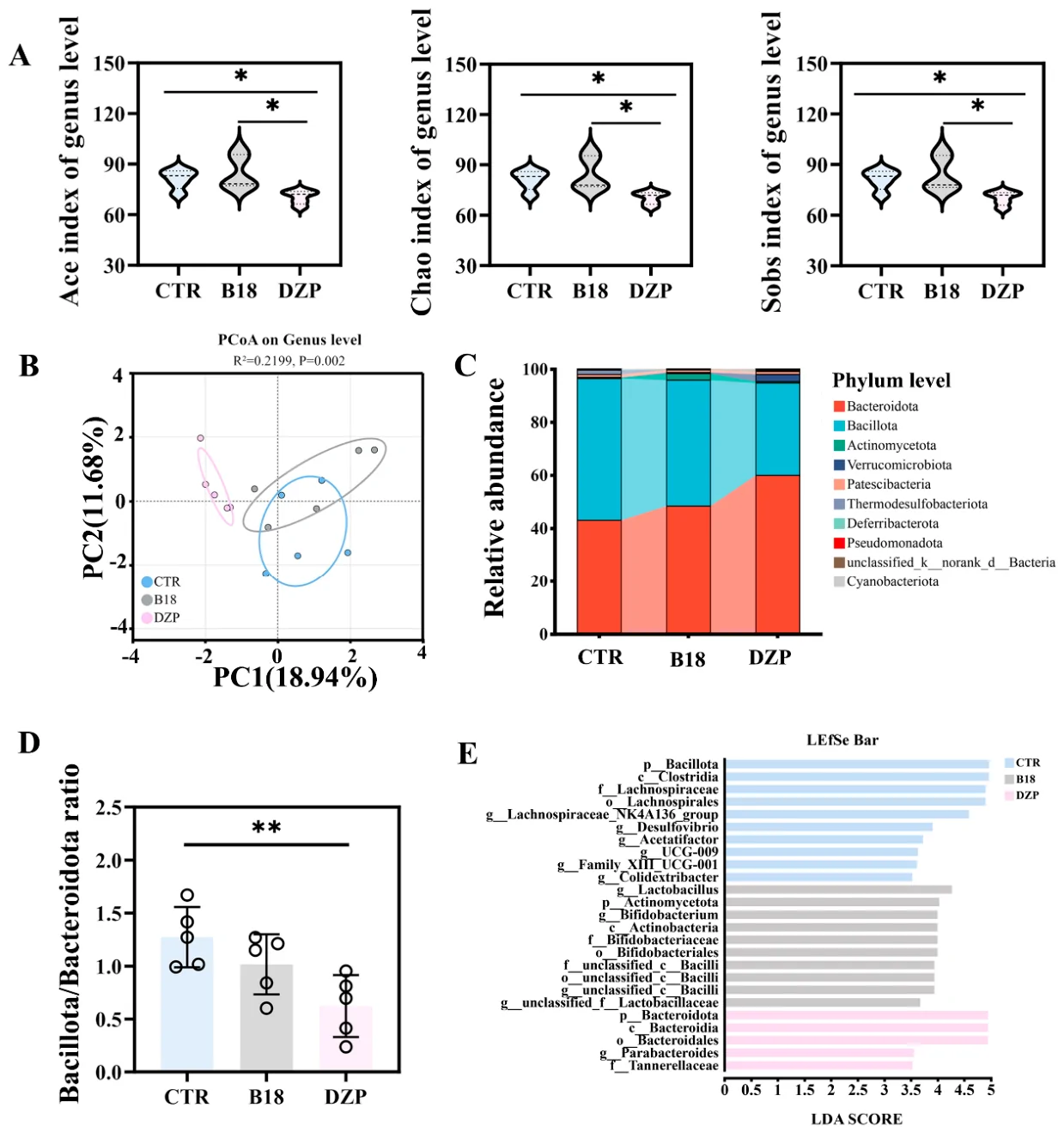
Gut microbiota composition in rats following *B. longum* B18 supplementation, as assessed by faecal 16S rRNA gene sequencing. (**A**) Alpha diversity indices (ACE, Chao1, and Sobs) at the genus level across the CTR, B18, and DZP groups. (**B**) Principal coordinate analysis (PCoA) based on binary Euclidean distances at the genus level. (**C**) Stacked bar chart showing the relative abundance of the dominant bacterial phyla in each group. (**D**) Bacillota/Bacteroidota ratio at the phylum level across the CTR, B18, and DZP groups. (**E**) LEfSe bar chart displaying differentially abundant taxa among the three groups (LDA score > 2). *n* = 5 rats per group. For panel (**A**), statistical significance was determined using the Kruskal–Wallis test followed by Dunn’s multiple-comparison test. For panel (**D**), statistical significance was determined by one-way ANOVA followed by Tukey’s post hoc test. * *p* < 0.05, ** *p* < 0.01.

To characterise gut microbial community composition following *B. longum* B18 supplementation, 16S rRNA gene sequencing was performed on faecal samples collected from the CTR, B18, and DZP groups. Assessment of within-sample microbial diversity based on the genus-level abundance table showed a marked decrease in the ACE, Chao1, and Sobs metrics in the DZP group relative to the CTR and B18 groups (*p* < 0.05; Figure 5A), suggesting that chronic diazepam administration markedly reduced gut microbial richness. Notably, the CTR and B18 groups showed comparable microbial richness across the ACE, Chao1, and Sobs indices, with no significant differences observed between the two groups. PCoA based on binary Euclidean distances revealed distinct clustering patterns among the CTR, B18, and DZP groups at the genus level (R^2^ = 0.2199, *p* = 0.002; Figure 5B). The DZP samples were relatively separated from the CTR and B18 groups, whereas partial overlap was observed between the CTR and B18 clusters. PERMDISP analysis showed no significant difference in multivariate dispersion among the three groups (F = 2.840, *p* = 0.101; Supplementary Figure S3), indicating no evidence of significant heterogeneity in within-group dispersion. Phylum-level profiling revealed Bacteroidota and Bacillota as the predominant phyla across all groups, together comprising the majority of the total gut microbiota (Figure 5C). The DZP group exhibited a pronounced decrease in the Bacillota/Bacteroidota ratio compared with the CTR (*p* < 0.01; Figure 5D), whereas the B18 group displayed an intermediate ratio without reaching statistical significance relative to CTR. This shift may reflect a phylum-level compositional change associated with prolonged benzodiazepine exposure, although its biological significance remains context dependent. Exploratory LEfSe analysis was performed to identify taxa potentially contributing to the differentiation among the three groups (LDA score > 2; Figure 5E). Given the small sample size (n = 5 per group) and the absence of additional multiple-testing correction, the LEfSe-derived taxa were interpreted as hypothesis-generating rather than confirmatory findings. Taxa contributing to the differentiation of the CTR mainly belonged to Bacillota, including *Clostridia*, *Lachnospiraceae*, *Lachnospirales*, *Lachnospiraceae_NK4A136_group*, *Desulfovibrio*, *Acetatifactor*, *UCG-009*, *Family_XIII_UCG-001*, *Colidextribacter*, and *Lactobacillus*. Taxa associated with the B18 group included Actinomycetota-affiliated taxa, including *Bifidobacterium*, *Actinobacteria*, *Bifidobacteriaceae*, and *Bifidobacteriales*, together with several unclassified taxa within *Bacilli* and *Lactobacillaceae*. Taxa associated with the DZP group included Bacteroidota-related taxa, including *Bacteroidia*, *Bacteroidales*, *Parabacteroides*, and *Tannerellaceae*. Collectively, these exploratory findings suggest group-associated differences in faecal microbial composition, with the B18 group showing a microbial profile more similar to that of the CTR than to that of the DZP group.

### 4.9. Differentially Abundant Gut Bacterial Genera and Their Associations with Tryptophan-Related Metabolism

**Figure 6:**
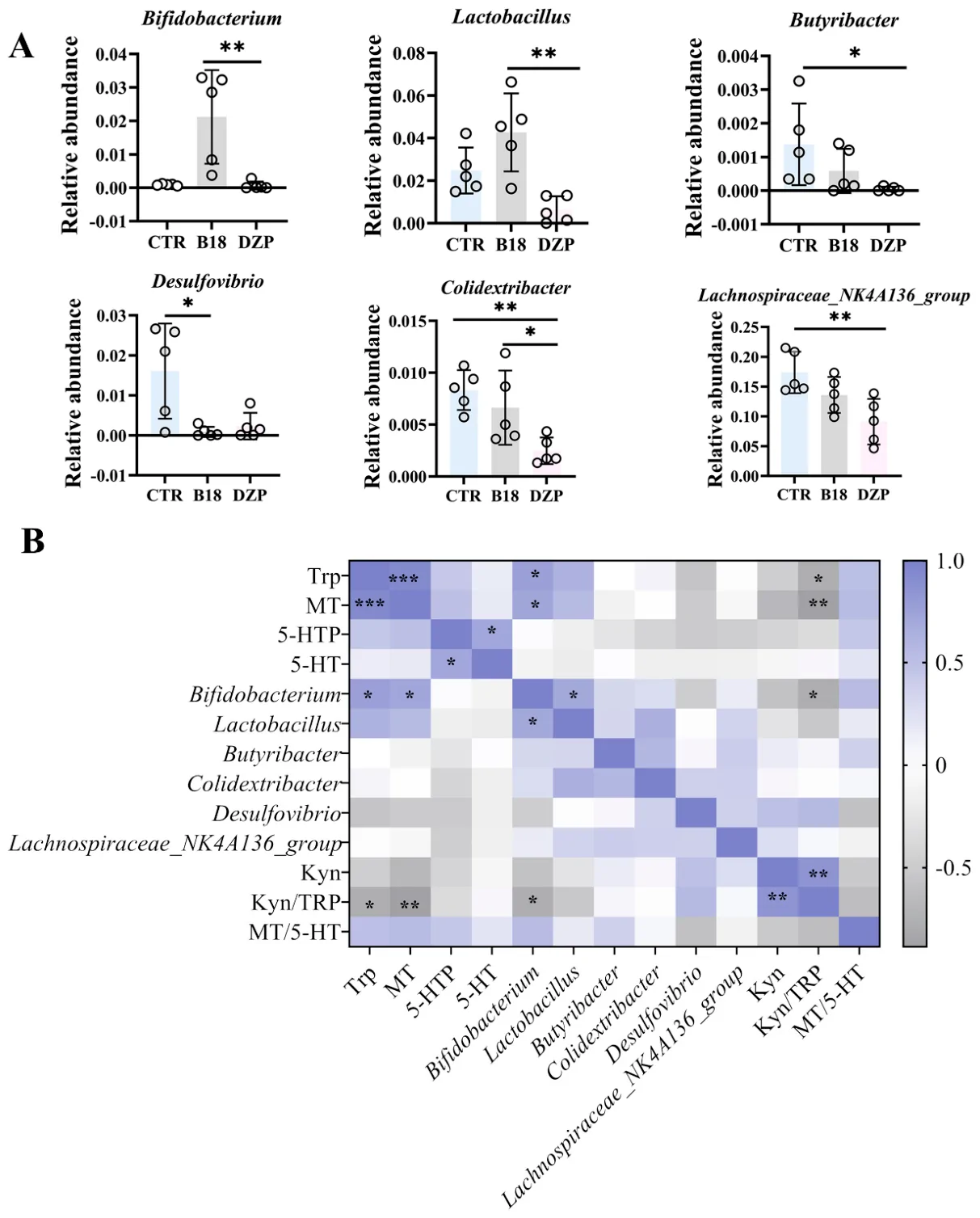
Differential gut microbial genera and their associations with tryptophan-related metabolites and metabolic ratios. (**A**) Relative abundances of the differential genera in the CTR, B18, and DZP groups (n = 5 per group). (**B**) Spearman correlation heatmap showing the associations between differential genera, tryptophan-related metabolites, and metabolic ratios. Correlations were calculated using paired microbiota and metabolite data obtained from the same animals in the CTR, B18, and DZP groups (n = 5 per group; n = 15 in total). *P*-values from the correlation analysis were adjusted for multiple testing using the Benjamini–Hochberg false discovery rate (FDR) procedure. Spearman correlation coefficients are represented by the colour scale. For panel (**A**), statistical significance was determined using the Kruskal–Wallis test followed by Dunn’s multiple-comparison test. * *p* < 0.05 and ** *p* < 0.01. For panel (**B**): * q < 0.05, ** q < 0.01, and *** q < 0.001.

To characterise genus-level differences among the CTR, B18, and DZP groups, key differentially abundant genera were analysed (Figure 6A). Compared with the DZP group, B18-treated animals exhibited higher abundances of *Bifidobacterium* and *Lactobacillus* (*p* < 0.01). In contrast, *Desulfovibrio* abundance was significantly lower in the B18 group than in the CTR (*p* < 0.05). *Colidextribacter* relative abundance was significantly higher in the CTR than in both the B18 (*p* < 0.01) and DZP groups (*p* < 0.05). Additionally, *Butyribacter* abundance was significantly lower in the DZP group than in the CTR (*p* < 0.05), and the *Lachnospiraceae* NK4A136 group also showed a significant reduction in the DZP group relative to the CTR (*p* < 0.01). Collectively, these findings indicate group-specific differences in the relative abundances of several bacterial genera among the CTR, B18, and DZP groups.

To further examine the relationships between gut microbiota compositional shifts and tryptophan-related metabolism, Spearman rank correlations were computed among differentially abundant bacterial genera, tryptophan-related metabolites (Trp, MT, 5-HTP, 5-HT, and Kyn), and two metabolic ratios (Kyn/Trp and MT/5-HT), followed by Benjamini–Hochberg false discovery rate (FDR) correction (Figure 6B). Faecal Trp levels showed a significant positive correlation with the relative abundance of *Bifidobacterium* (q < 0.05) and were strongly positively associated with MT levels (q < 0.001), while showing an inverse association with the Kyn/Trp ratio (q < 0.05). Faecal MT levels were likewise positively associated with *Bifidobacterium* abundance (q < 0.05) and inversely associated with the Kyn/Trp ratio (q < 0.01), highlighting a coordinated relationship among *Bifidobacterium* abundance, Trp availability, and MT levels. Notably, the Kyn/Trp ratio was inversely associated with *Bifidobacterium* abundance (q < 0.05) and positively associated with Kyn levels (q < 0.01), whereas *Bifidobacterium* abundance was positively correlated with *Lactobacillus* abundance (q < 0.05). In parallel, 5-HTP levels were positively correlated with 5-HT levels (q < 0.05), although neither metabolite showed significant associations with the differential bacterial genera after FDR correction. Collectively, the FDR-adjusted correlation analysis identified exploratory associations between *Bifidobacterium* abundance and variation in faecal Trp, MT, and the Kyn/Trp ratio, supporting an association between gut microbial composition and tryptophan–melatonin metabolism. 

## 5. Discussion

The present study demonstrates that *B. longum* B18 supplementation shortened barbital sodium-induced sleep latency and prolonged pentobarbital sodium-induced sleep duration, accompanied by increased Trp and MT concentrations across faeces, plasma, and hippocampus, reduced Kyn/Trp ratios, and elevated hippocampal AANAT levels. In parallel, exploratory FDR-adjusted correlation analyses identified associations of *Bifidobacterium* abundance with faecal Trp, MT, and the Kyn/Trp ratio. Taken together, these findings identify a coordinated relationship between B18 supplementation, tryptophan–melatonin metabolism, gut microbial composition, and pharmacologically induced sleep parameters, while the causal contributions of specific microbial taxa and host metabolic pathways remain to be established.

The in vitro safety and probiotic-related properties of B18 define, to a considerable extent, the likelihood that this strain can survive oral delivery and interact with the host in a biologically meaningful manner. The non-haemolytic phenotype and favourable antibiotic susceptibility profile provide preliminary evidence for the biosafety of B18, supporting its further evaluation as a probiotic candidate for functional food development [34,35]. The relatively high survival following sequential exposure to simulated gastric and intestinal fluids indicates substantial gastrointestinal stress tolerance, essential for viable cell delivery to the intestinal tract [36,37]. Additionally, the relatively high hydrophobicity, strong auto-aggregation, and co-aggregation ability with *Escherichia coli* suggest that B18 may possess surface characteristics favorable for interaction with the intestinal mucosal environment [38,39,40]. Although these phenotypes cannot be directly equated with definitive colonisation in vivo, they are widely regarded as indicators of adhesion-related potential and may increase the opportunity for host–microbe interaction [41,42]. Moreover, co-aggregation with potentially undesirable bacteria may provide an additional ecological advantage by contributing to competitive exclusion and stabilisation of the intestinal microenvironment [43]. Collectively, these characteristics support the suitability of B18 for oral probiotic evaluation and provide relevant biological context for interpreting the effects on pharmacologically induced sleep parameters observed. 

*B. longum* B18 significantly shortened barbital sodium-induced sleep latency and prolonged pentobarbital sodium-induced sleep duration under suprathreshold conditions, but did not significantly increase sleep onset rate under subthreshold conditions (Supplementary Section S2.3 and Table S3) or produce an independent sleep-promoting effect without pharmacological induction (Supplementary Section S2.4 and Table S4). These results indicate that B18 may not act as a classical sedative–hypnotic agent that directly induces sleep; rather, B18 may modulate sleep-related parameters under pharmacological induction, as reflected by shortened sleep latency and prolonged sleep duration. Such a mode of action is consistent with the broader concept that microbe-based interventions modulate sleep through regulation of host metabolism, neurotransmitter precursor availability, and gut–brain axis signalling, rather than through direct central nervous system depression [44,45,46]. Consistent with this view, recent studies have demonstrated that probiotic interventions, including *Lactobacillus plantarum* P8 combined with traditional Chinese medicine, improve sleep-related parameters in association with alterations in gut microbiota composition, SCFA-related metabolism, and sleep-related neurochemical signalling [47]. These findings further support the possibility that microbiota-associated metabolic regulation may represent one pathway involved in sleep modulation.

Targeted quantitative analysis revealed that B18 intervention consistently and significantly elevated Trp and MT concentrations across faeces, plasma, and hippocampus, while leaving 5-HTP and 5-HT levels statistically unchanged. This metabolic pattern is particularly informative because it argues against a simple linear activation of the classical Trp → 5-HTP → 5-HT → MT biosynthetic cascade and instead may reflect differential regulation of multiple steps within tryptophan-related metabolism, as discussed below.

The absence of significant changes in 5-HTP and 5-HT, despite marked elevations in both upstream Trp and downstream MT across faeces, plasma, and hippocampus, may be partially explained by coordinated alterations at multiple points in tryptophan-related metabolism. First, B18 supplementation was associated with reduced Kyn/Trp ratios across all three compartments. In faeces and hippocampus, the lower Kyn/Trp ratios were accompanied by reduced Kyn concentrations, a pattern consistent with altered kynurenine-related tryptophan metabolism and potentially greater Trp availability. In plasma, however, the reduced Kyn/Trp ratio occurred alongside increased Trp concentrations, while Kyn concentrations remained statistically unchanged, indicating that the metabolic basis of the ratio change may differ among compartments. Second, the elevated hippocampal AANAT levels observed following B18 supplementation, together with increased MT concentrations, suggest enhanced involvement of AANAT-associated melatonin metabolism. Greater Trp availability may provide additional substrate for serotonergic metabolism, while increased downstream utilisation of serotonergic intermediates could contribute to MT accumulation without necessarily altering steady-state 5-HT concentrations. Third, the gastrointestinal tract is recognised as an important extra-pineal source of melatonin that can operate independently of the canonical pineal serotonergic pathway [48]. Previous studies have also reported melatonin-related metabolic properties in selected strains of *Bifidobacterium* and *Lactobacillus* [49,50]. In the present study, the relative abundances of these genera were higher in the B18 group than in the DZP group, and *Bifidobacterium* abundance was positively associated with faecal MT concentrations, raising the possibility that intestinal microbial metabolism may represent an additional contributor to the faecal MT profile observed following B18 supplementation. Collectively, these findings suggest that the characteristic increase in Trp and MT concentrations in the absence of corresponding changes in 5-HTP and 5-HT may reflect coordinated alterations in kynurenine-related and melatonin-related metabolism rather than simple activation of a single linear pathway. The significant associations of faecal Trp and MT concentrations with pharmacologically induced sleep parameters further link these metabolic alterations to the sleep parameters observed following B18 supplementation.

Under basal physiological conditions, dietary Trp is predominantly catabolised through the kynurenine pathway, which is regulated by several enzymes, including tryptophan 2,3-dioxygenase (TDO) and indoleamine 2,3-dioxygenase 1 (IDO1), leading to Kyn formation and downstream metabolite production [51]. IDO1 expression can be induced by inflammatory cytokines such as IFN-γ and TNF-α and may be associated with NF-κB inflammatory signalling [52], Accordingly, the changes in kynurenine-related metabolism observed following B18 supplementation may be associated with alterations in the intestinal microbial and inflammatory milieu. In this context, the lower abundance of *Desulfovibrio* observed in the B18 group relative to the CTR may be relevant, as members of this genus have been associated with LPS- and hydrogen sulfide-related pro-inflammatory signalling [53,54]. Previous studies have also reported that selected *Bifidobacterium* strains can influence IDO1 expression and kynurenine pathway activity [20], providing biological context for the association between *Bifidobacterium* abundance and kynurenine-related metabolism observed in the present study. Alterations in kynurenine-related metabolism may also have broader neurobiological relevance, as downstream metabolites such as QUIN have been implicated in neuronal function and sleep regulation [55,56]. However, these relationships remain associative, and direct regulation of IDO1 or inflammatory signalling by B18 was not examined in the present study.

In parallel with the alterations in kynurenine-related tryptophan metabolism, B18 supplementation was also associated with changes in melatonin-related metabolism. The higher hippocampal AANAT levels, together with increased hippocampal MT concentrations, support a potential involvement of AANAT-associated melatonin metabolism, although the underlying regulatory mechanisms remain to be established. Several processes may contribute to the higher hippocampal AANAT levels observed following B18 supplementation. First, the increased Trp availability associated with B18 treatment may provide greater substrate availability for downstream serotonergic–melatonin metabolism. Second, members of *Bifidobacterium* and *Lactobacillus* have been associated with acetate and lactate production and with cross-feeding interactions involving butyrate-producing bacteria [57,58]. Butyrate has been reported to promote CREB-dependent AANAT transcription [59], suggesting that microbial metabolic interactions may represent one potential route through which AANAT regulation is influenced. Third, inflammatory signalling may also be relevant, as the lower abundance of *Desulfovibrio* in the B18 group than in the CTR may be associated with a less pro-inflammatory intestinal milieu, while IL-1β [60] and TNF-α [61] have been reported to suppress AANAT expression under inflammatory conditions. However, neither butyrate levels nor inflammatory markers were directly assessed in the present study. Collectively, these observations suggest that substrate availability, microbial metabolic interactions, and inflammatory signalling may each contribute to the altered AANAT-associated melatonin metabolism observed following B18 supplementation.

It is noteworthy that the hippocampal MT/5-HT ratio did not differ significantly between the B18 and DZP groups, despite the significant increases observed in faeces and plasma following B18 supplementation. This pattern suggests that the effects of B18 and diazepam on hippocampal melatonin-related metabolism may not be identical to those observed in peripheral compartments. Given that diazepam primarily acts through GABAergic neurotransmission, its influence on hippocampal melatonin-related metabolism may involve mechanisms distinct from those associated with B18 supplementation. In the B18 group, the higher hippocampal AANAT levels, together with the increased hippocampal MT concentration, support a potential involvement of AANAT in the altered melatonin-related metabolic profile.

The gut microbiota differences observed among the treatment groups may be associated with the changes in tryptophan-related metabolism described above. At the genus level, the observed differences were comparator-specific, with higher relative abundances of *Bifidobacterium* and *Lactobacillus* in the B18 group than in the DZP group and lower *Desulfovibrio* abundance than in the CTR. Among the FDR-adjusted associations, *Bifidobacterium* abundance showed notable associations with the tryptophan–melatonin metabolic profile, being associated with faecal Trp, MT, and the Kyn/Trp ratio. These findings suggest that variation in *Bifidobacterium* abundance accompanied the metabolic changes observed following B18 supplementation, although the present correlations do not establish a causal role for this genus in regulating tryptophan-related metabolism.

The DZP group also exhibited a lower Bacillota/Bacteroidota ratio than the CTR, together with lower abundances of *Butyribacter* and the *Lachnospiraceae* NK4A136 group relative to CTR. These compositional features are consistent with previous evidence that benzodiazepine exposure is associated with alterations in gut microbial community structure [62]. Taken together with the comparable microbial richness observed between the B18 and CTR, these findings further indicate that B18 and diazepam were associated with distinct patterns of gut microbial composition.

The present study employed barbital- and pentobarbital-induced rat sleep models to evaluate the effects of *B. longum* B18 on pharmacologically induced sleep parameters, rather than directly assessing spontaneous sleep or sleep architecture. Because these hypnotic agents exert strong pharmacological effects on central sleep-regulatory processes, this experimental paradigm may reduce sensitivity to more subtle B18-associated effects on physiological sleep. Future studies incorporating EEG/EMG recordings are therefore warranted to determine whether B18 influences specific sleep stages and the organisation of physiological sleep. In addition, although the integrated microbiota and metabolomic analyses revealed associations between gut microbial composition and tryptophan-related metabolism, the direction and functional significance of these relationships require further investigation. The LEfSe analysis was exploratory because of the small microbiota sample size (*n* = 5 per group), and no additional multiple-testing correction was applied; therefore, the identified taxonomic features require validation in larger independent cohorts. Faecal microbiota transplantation experiments in germ-free or antibiotic-treated animals may help determine whether gut microbial changes contribute to the metabolic alterations and pharmacologically induced sleep parameters associated with B18 supplementation. Moreover, the V3–V4 16S rRNA sequencing approach used here does not allow strain-level identification; therefore, future studies incorporating strain-specific qPCR or other targeted approaches would be valuable for tracking B18 in the intestinal tract. Only male rats were included in the present study, and potential sex-dependent responses to B18 therefore remain to be explored. Finally, the extent to which these findings translate to clinically defined sleep disorders requires further investigation.

## 6. Conclusions

The present study indicates that *Bifidobacterium longum* B18 is a promising probiotic candidate with favourable functional properties and may modulate pharmacologically induced sleep parameters in rats. B18 supplementation was associated with alterations in tryptophan-related metabolism, characterised by reduced Kyn/Trp ratios, increased Trp and MT concentrations, and elevated hippocampal AANAT levels. Gut microbiota analysis further revealed treatment-associated differences in microbial composition, including higher abundances of *Bifidobacterium* and *Lactobacillus* in the B18 group than in the DZP group and lower *Desulfovibrio* abundance than in the CTR. Collectively, these findings suggest that the effects of B18 on pharmacologically induced sleep parameters are associated with changes in tryptophan–melatonin metabolism and gut microbial composition, supporting further evaluation of B18 as a probiotic candidate for sleep-related functional applications.

## Figures and Tables

**Table 1:** Antimicrobial susceptibility of *Bifidobacterium longum* B18 determined by broth microdilution.

Antibiotic	Concentration Range (μg/mL)	MIC of B18 (μg/mL)	EFSA Cut-Off (μg/mL)	Interpretation
Ampicillin	0.125–16	1	2	susceptible
Vancomycin	0.125–16	0.5	2	susceptible
Gentamicin	4–256	16	64	susceptible
Streptomycin	8–512	32	128	susceptible
Erythromycin	0.0625–8	0.5	1	susceptible
Clindamycin	0.0625–8	0.25	1	susceptible
Tetracycline	0.5–64	2	8	susceptible
Chloramphenicol	0.25–32	2	4	susceptible

**Table 2:** Cell surface hydrophobicity of *B. longum* B18 determined using xylene.

Strain Name	*B. longum* B18
Hydrophobicity (%)	62.86 ± 4.45

## Data Availability

The raw 16S rRNA gene sequencing data generated in this study have been deposited in the NCBI under BioProject accession number PRJNA1496274. Other data supporting the findings of this study are available from the corresponding authors upon reasonable request.

## Supplementary Materials

The following supporting information can be downloaded at: https://www.mdpi.com/article/10.3390/foods15183230/s1, References [63,64,65] are cited in the Supplementary Materials.
