# Supplementary material for: Bifidobacterium longum B18 Modulates Pharmacologically Induced Sleep Parameters in Rats in Association with Altered Tryptophan–Melatonin Metabolism
Source: Foods. 2026 Sep 12;15(18):3230. doi: 10.3390/foods15183230 (PMC13606917; doi:10.3390/foods15183230)
Supplement: Supplementary file 1 [file foods-15-03230-s001.zip › foods-4504384-supplementary.pdf]

Supplementary materials

Supplementary Methods

S1.1. Cell surface hydrophobicity of B. longum B18

Bacterial cell surface hydrophobicity was assessed using a modified version of a previously reported method [1]. *B. longum* B18 was cultured and harvested as described in Section 2.3 and resuspended in physiological saline to an OD_{600} of 0.8–1.0. An aliquot (3 mL) of this suspension was mixed with 1 mL xylene, equilibrated at 37°C for 10 min, vortexed, and incubated at 37°C for 3 h to allow phase separation. The aqueous phase was then carefully collected for OD_{600} measurement. All assays were performed in triplicate, and hydrophobicity was calculated as follows:

$$K_4=\frac{C_0-C_t}{C_0}\times 100\%$$

where K_4 represents the hydrophobicity of B18 (%), and C_0 and C_t represent the OD_{600} values of the bacterial suspension before and after mixing with xylene, respectively.

S1.2. Sleep assessment criteria and definitions

The righting reflex was used as the criterion for assessing sleep status in rats. The righting reflex refers to the physiological ability of a conscious animal to rapidly return to a normal prone position after being placed on its back with all four limbs facing upward. After drug administration, each rat was gently placed in the supine position. If the animal failed to return spontaneously to the normal prone position within 1 min, and this result was consistently observed in three consecutive assessments, the righting reflex was considered lost, indicating that the animal had entered sleep. Recovery of the righting reflex was defined as the ability of the rat to regain the normal prone position. Sleep latency was defined as the time from drug injection to the loss of the righting reflex, and sleep duration was defined as the time from the loss to the recovery of the righting reflex.

S1.3. Randomisation and Blinded Outcome Assessment

After the one-week acclimatisation period, each rat was assigned a unique identification number by ear tagging and randomly allocated to an experimental group using a computer-generated random-number sequence. Randomisation was performed independently for each experimental batch. Oral gavage and drug administration were conducted by the same trained investigator. Sleep latency and sleep duration were assessed according to predefined standardised criteria by trained investigators who were blinded to group allocation throughout outcome assessment. No formal a priori sample-size calculation was performed. The group size was selected based on previous experience with comparable rat sleep experiments and practical considerations.

S1.4. Direct sleep test

Twenty rats were randomly divided into two groups (n = 10 per group). The normal control group (CTR) received 1 mL of saline, and the probiotic group received 1 mL of a probiotic suspension (1×10^9 CFU/mL), both administered by oral gavage once daily for 30 consecutive days. At 30 min after the final administration, sleep latency and sleep duration were recorded in each group.

S1.5. Determination of the minimum supra-threshold and maximum sub-threshold doses of sodium pentobarbital

To determine the minimum supra-threshold dose of sodium pentobarbital (PB) that induced sleep in 100% of rats and the maximum sub-threshold dose that induced sleep in approximately 10%–20% of rats, rats in each group (n = 10) were intraperitoneally administered PB at different doses (30, 35, 40, 45, 50, 55, and 60 mg/kg). The number of rats that entered sleep within 30 min after injection was recorded.

S1.6. Determination of the minimum supra-threshold and maximum sub-threshold doses of sodium barbital

To determine the minimum supra-threshold dose of sodium barbital (PhB) that induced

sleep in 100% of rats and the maximum sub-threshold dose that induced sleep in approximately 10%–20% of rats, rats in each group (n = 10) were intraperitoneally administered different doses of PhB (200, 250, 280, 300, 320, and 340 mg/kg). The number of rats that entered the sleep state within 30 min after injection was recorded.

S1.7. Effect of the B. longum B18 on sleep onset rate in rats

Thirty rats were randomly assigned to three groups (n = 10 per group). The normal control group received an equal volume of saline (1 mL/rat) by oral gavage, the positive control group received diazepam (3 mg/kg, 1 mL/rat), and the probiotic group received a *B. longum* B18 suspension (1 × 10^9 CFU/mL, 1 mL/rat). All treatments were administered once daily for 30 consecutive days. At 30 min after the final administration, all rats were intraperitoneally injected with the maximum sub-threshold dose of pentobarbital sodium. The number of sleeping rats within 30 min was recorded, and the sleep onset rate (%) was calculated to assess the synergistic effect of the probiotic on pentobarbital sodium-induced sleep.

S1.8. Composition of the modified MRS (mMRS) medium used for the cultivation of Bifidobacterium longum B18

The modified de Man, Rogosa and Sharpe (mMRS) medium used for the cultivation of *B. longum* B18 was prepared according to the composition shown in Table S13.

1.9. Accuracy and Precision Evaluation of the UHPLC–MS/MS Method

The accuracy and precision of the UHPLC–MS/MS method were evaluated using matrix-spiked quality control (QC) samples at low, medium, and high concentration levels. Biological matrices (faeces, plasma, and hippocampal homogenates) were

spiked with known amounts of the target analytes prior to sample extraction. Corresponding unspiked matrix samples were processed and analysed in parallel to determine the endogenous concentrations of the target analytes. After sample preparation and UHPLC–MS/MS analysis, the endogenous concentrations determined in the unspiked matrices were subtracted from the measured concentrations of the corresponding spiked samples.

S1.10. Metabolite quantification and normalization

Metabolite concentrations in the reconstituted extracts were determined from the corresponding calibration curves after internal-standard correction and were initially expressed as ng/mL. For fecal and hippocampal samples, the concentrations were converted to the amount per unit sample weight according to the initial sample mass and final reconstitution volume and were expressed as ng/g. For plasma samples, concentrations were normalized to the initial plasma volume and expressed as ng/mL. The concentrations were calculated as follows:

$$C_{\text{feces/hippocampus}} = \frac{C_{\text{extract}} \times V_{reconstitution}}{m_{\text{sample}}}$$

$$C_{\text{plasma}} = \frac{C_{\text{extract}} \times V_{reconstitution}}{V_{\text{plasma}}}$$

Where C_{extract} represents the metabolite concentration measured in the reconstituted extract (ng/mL), $V_{reconstitution}$ is the final reconstitution volume (0.100 mL), m_{sample} is the initial mass of the fecal or hippocampal sample (0.060 g), and V_{plasma} is the initial plasma volume (0.100 mL).

S1.11. Experimental Diet Composition

Rats were provided ad libitum with SPF maintenance rodent chow supplied by the

Guangdong Medical Laboratory Animal Center (Guangdong, China). According to the supplier's typical analysis, the diet contained 185 g/kg protein, 46 g/kg fat, 589 g/kg carbohydrate, 32 g/kg crude fiber, 68 g/kg ash, 12.8 g/kg calcium, 9.2 g/kg phosphorus, and 2.0 g/kg tryptophan (0.20%, w/w).

S1.12. Bioinformatic Processing and Statistical Analysis of 16S rRNA Gene Sequencing Data

Raw paired-end reads were demultiplexed according to sample-specific barcode sequences. Sequence quality filtering was performed using fastp (version 0.19.6), and paired-end reads were merged using FLASH (version 1.2.11). During quality control, low-quality bases with a quality score below Q20 were trimmed using a sliding-window approach. Reads shorter than 50 bp after trimming and reads containing ambiguous nucleotides (N) were removed. Paired-end reads were subsequently merged based on overlapping regions, with a minimum overlap length of 10 bp and a maximum mismatch ratio of 0.2.

High-quality sequences were processed using the DADA2 denoising algorithm implemented in QIIME2 (2024 release) to generate amplicon sequence variants (ASVs). Taxonomic assignment of ASVs was performed using the classify-sklearn method based on a Naive Bayes classifier against the SILVA 16S rRNA gene database (release 138.2, 16S bacteria) with a classification confidence threshold of 0.7. ASVs assigned as chloroplast or mitochondrial sequences were removed before downstream analyses. Low-abundance ASVs with a relative abundance below 0.001 across all sequences were filtered out to reduce the influence of rare features.

To minimize the effects of differences in sequencing depth among samples, the ASV abundance table was rarefied according to the minimum sequencing depth (35023) among samples before diversity analyses. The rarefied ASV table was subsequently used for microbial community composition analysis and diversity analyses.

Alpha-diversity indices, including ACE, Chao1, and Sobs, were calculated at the genus level based on the rarefied abundance table. Differences in alpha-diversity indices among experimental groups were evaluated using the Kruskal–Wallis rank-sum test, followed by Dunn's post hoc test for pairwise comparisons. A p value < 0.05 was considered statistically significant.

Beta-diversity analysis was performed at the genus level using binary Euclidean distances. Principal coordinate analysis (PCoA) was used to visualise differences in microbial community composition among the CTR, B18, and DZP groups. Differences in community composition among groups were assessed using permutational multivariate analysis of variance (PERMANOVA; Adonis) with 999 permutations. Homogeneity of multivariate dispersion among groups was further evaluated using PERMDISP based on the same binary Euclidean distance metric, with 999 permutations.

Differentially abundant microbial taxa among experimental groups were identified using the linear discriminant analysis effect size (LEfSe) method. Taxa with an LDA score >2 were considered discriminative features. Considering the relatively small sample size (n = 5 per group), LEfSe analysis was regarded as exploratory, and identified taxa were interpreted as potential microbial signatures associated with different experimental conditions.

PCR amplification negative controls containing nuclease-free water instead of template DNA were included to monitor potential reagent contamination. No detectable amplification products were observed in negative controls.

S1.13. Preparation and Standardisation of B18 Suspension for Animal Administration

For the animal experiments, B18 cells were cultured to the late logarithmic phase and harvested by centrifugation (5000 × *g*, 10 min, 4°C). The cells were washed twice with PBS and resuspended in sterile physiological saline. Based on a previously established relationship between OD_{600} and bacterial concentration (CFU/mL), the OD_{600} of the bacterial suspension was adjusted to the predetermined value corresponding to approximately 1×10^9 CFU/mL. Viable bacterial counts were further verified by serial dilution and plate counting to confirm the actual CFU concentration of the administered suspension. Fresh B18 suspensions were prepared for administration, and each rat received 1 mL by oral gavage once daily for 35 consecutive days, corresponding to approximately 1×10^9 viable CFU per animal per day. The dose was selected with reference to previous rodent probiotic intervention studies using comparable doses [2, 3].

S1.14. Calibration Curves, Linearity, and Limits of Detection and Quantification

Calibration curves were established separately for each target analyte in faecal, plasma, and hippocampal matrices using the internal standard method. Calibration curves were constructed by plotting the analyte-to-IS peak-area ratio (y) against the corresponding analyte concentration (x) and were fitted by linear regression. Linearity was evaluated using the coefficient of determination (R^2). The limits of detection (LOD) and quantification (LOQ) were calculated as 3.3σ/S and 10σ/S, respectively, where σ represents the standard deviation of the analytical response and S represents the slope of the corresponding calibration curve. As the calculated LOQ values were below the lowest standards of the corresponding calibration curves, the calculated LOD and LOQ values were used as estimates of method sensitivity, whereas the lowest calibration

standard was adopted as the lower limit of the quantitative working range.

S1.15. Extraction Recovery and Matrix Effects

Extraction recovery and matrix effects were evaluated in faecal, plasma, and hippocampal matrices at low, medium, and high QC concentration levels. Although the target metabolites were quantified using an internal standard (IS) method, extraction recovery and matrix effects were evaluated on the basis of the peak areas of the target analytes.

Four sets of samples were prepared for each analyte and biological matrix. Set A consisted of neat standard solutions containing the target analyte at the corresponding concentration. For set B, biological matrix samples were first subjected to the complete extraction procedure, after which the target analyte was added to the resulting matrix extract (post-extraction spiked samples). For set D, the target analyte was spiked into the biological matrix before extraction and subsequently subjected to the complete extraction procedure (pre-extraction spiked samples). Set E consisted of corresponding unspiked biological matrix samples that underwent the same extraction procedure and was used to determine the endogenous analyte response. The internal standard was added according to the same procedure used for quantitative UHPLC–MS/MS analysis. Each condition was analysed in six replicates (n = 6).

The analyte peak areas obtained from sets A, B, D, and E were denoted as A_A, A_B, A_D, and A_E, respectively. Because the target metabolites were endogenously present in the biological matrices, the endogenous analyte peak area measured in set E was subtracted from the corresponding peak areas of the spiked matrix samples before calculation.

Extraction recovery was calculated as:

$$\text{Recovery (\%)} = \frac{A_D - A_E}{A_B - A_E} \times 100$$

Matrix effects (ME) were calculated as:

$$\text{ME (\%)} = \frac{A_B - A_E}{A_A} \times 100$$

where A_A represents the analyte peak area in the neat standard solution (set A), A_B represents the analyte peak area in the post-extraction spiked matrix sample (set B), A_D represents the analyte peak area in the pre-extraction spiked matrix sample (set D), and

A_E represents the endogenous analyte peak area in the corresponding unspiked matrix sample (set E).

*S*2. Supplementary Results

S2.1. Determination of the minimum supra-threshold and maximum sub-threshold doses of pentobarbital sodium

To determine the appropriate dose range of sodium pentobarbital for sleep induction in rats, sleep onset under different dosing conditions was evaluated. As shown in Table S1, pentobarbital sodium at 50 mg/kg induced sleep in 100% of rats and was therefore defined as the minimum supra-threshold dose. When the dose was reduced to 35 mg/kg, only approximately 10%–20% of rats entered the sleep state; accordingly, 35 mg/kg was defined as the maximum sub-threshold dose for subsequent experiments.

Table S1. Minimum supra-threshold dose and maximum sub-threshold dose of pentobarbital sodium

Concentration (mg/kg)	30	35	40	45	50	55	60
Number of rats	10	10	10	10	10	10	10
Number of sleeping rats	0	2	5	8	10	10	10
Sleep onset rate (%)	0	20	50	80	100	100	100

S2.2. Determination of the minimum supra-threshold and maximum sub-threshold doses of barbital sodium

As shown in Table S2, barbital sodium at 300 mg/kg induced sleep in 100% of rats and was therefore defined as the minimum supra-threshold dose. When the dose was reduced to 250 mg/kg, only approximately 10%–20% of rats entered the sleep state; accordingly, 250 mg/kg was defined as the maximum sub-threshold dose for subsequent

experiments.

Table S2. Minimum supra-threshold dose and maximum sub-threshold dose of sodium barbital

Concentration (mg/kg)	200	250	280	300	320	340
Number of rats	10	10	10	10	10	10
Number of sleeping rats	0	2	5	10	10	10
Sleep onset rate (%)	0	20	50	100	100	100

S2.3. Effect of B18 on sleep onset rate in rats under the maximum sub-threshold dose of pentobarbital sodium

As shown in Table S3, 30 min after probiotic gavage, rats in each group were intraperitoneally injected with pentobarbital sodium at the maximum subthreshold dose (35 mg/kg). During the 30 min observation period, B18 intervention did not significantly affect the sleep onset rate of rats compared with the blank control group ($P > 0.05$).

Table S3 Effect of B18 on the sleep onset rate of rats under the maximum sub-threshold dose of pentobarbital sodium

Group	CTR	B18	DZP
Pentobarbital sodium dose (mg/kg)	35	35	35
Total number of rats	10	10	10
Number of sleeping rats	2	3	6
Sleep onset rate (%)	20	30	60

S2.4. B18 did not exert a direct sleep-promoting effect in rats

As shown in Table S4, following probiotic administration by oral gavage, *Bifidobacterium longum* B18 did not exert a significant direct sleep-promoting effect in rats ($P > 0.05$).

Table S4 Direct sleep-promoting effect of *B. longum* B18 in rats

Group	CTR	B18
Total number of rats (n)	10	10
Number of sleeping rats (n)	0	0
Sleep onset rate (%)	0	0

S2.5. Effects of B. longum B18 on 5-HTP and 5-HIAA Levels in the Plasma, Feces, and Hippocampus of Rats

As shown in Figure S1, neither 5-hydroxyindoleacetic acid (5-HIAA) nor 5-hydroxytryptophan (5-HTP) concentrations differed significantly among the CTR, B18, and DZP groups across fecal, plasma, and hippocampal compartments ($P > 0.05$), suggesting that B18 supplementation did not perturb the upstream hydroxylation of tryptophan to 5-HTP or the downstream oxidative deamination of serotonin to 5-HIAA. These findings indicate that the observed changes in tryptophan and melatonin metabolism were not accompanied by alterations in serotonin turnover, consistent with a selective modulation of tryptophan metabolic flux toward the melatonin biosynthetic branch rather than a global perturbation of the serotonergic pathway.

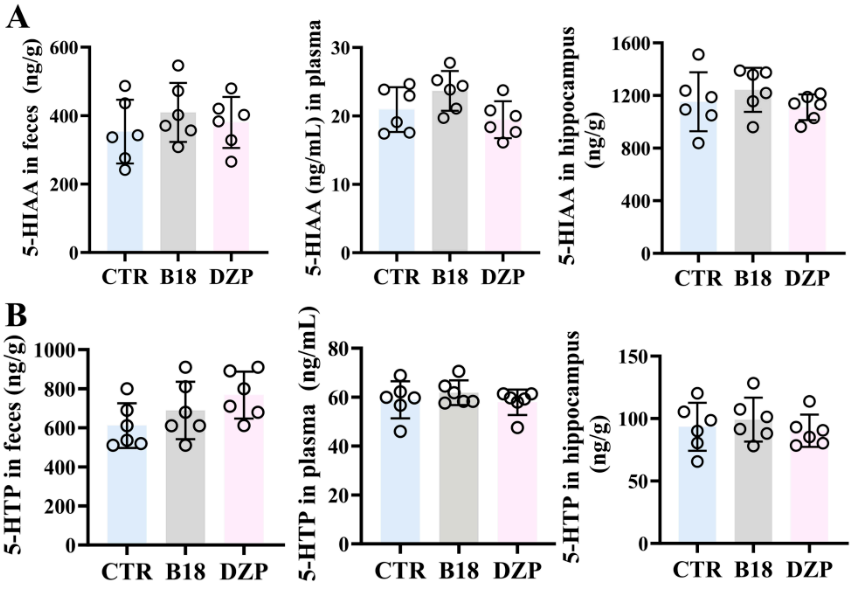

Figure S1. Effects of *B. longum* B18 on the levels of 5-HIAA and 5-HTP in the feces,

plasma, and hippocampus of rats. (A) 5-HIAA; (B) 5-HTP. n = 6 rats per group. Data are presented as mean ± SD. Statistical significance was determined by one-way ANOVA followed by Tukey's post hoc test. *$P < 0.05$, **$P < 0.01$.

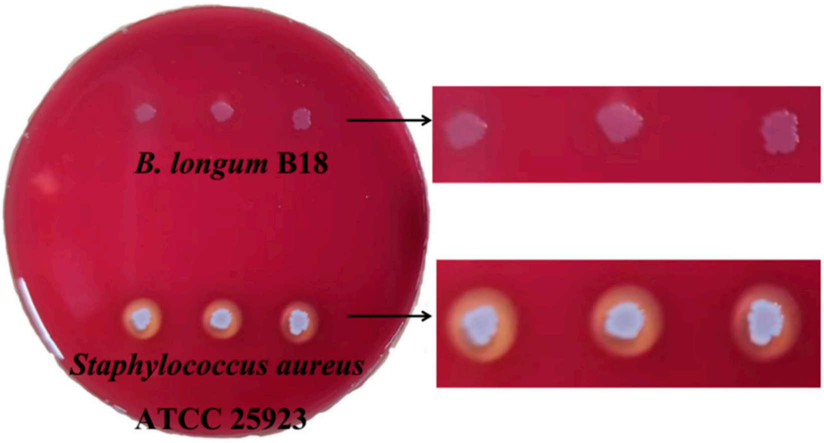

Figure S2. Hemolytic activity of B18 using *Staphylococcus aureus* ATCC 25923 as a positive control.

Table S5. Viable counts and survival of *B. longum* B18 after exposure to simulated gastric fluid

Replicate	Gastric fluid, 0 h (CFU)	Gastric fluid, 3 h (CFU)	Survival rate after 3 h of gastric fluid exposure (%)
1	1.00×10^8	8.545×10^7	85.45
2	1.00×10^8	7.875×10^7	78.75
3	1.00×10^8	8.745×10^7	87.45

Table S6. Viable counts and survival of *B. longum* B18 after exposure to simulated intestinal fluid

Replicate	Intestinal fluid, 0 h (CFU)	Intestinal fluid, 3 h (CFU)	Survival rate after 3 h of intestinal fluid exposure (%)
1	8.545×10^7	6.371×10^7	74.56
2	7.875×10^7	5.932×10^7	75.33
3	8.745×10^7	7.035×10^7	80.45

Table S7. UHPLC–MS/MS instrument and ion-source parameters

Parameter	Setting
UHPLC system	Ultimate 3000 UHPLC system (Thermo Fisher Scientific, USA)
Mass spectrometer	Q Exactive Focus Orbitrap mass spectrometer (Thermo Fisher Scientific, USA)
Ionization source	HESI (heated electrospray ionization)
Ionization mode	ESI+ (positive electrospray ionization mode)
Acquisition mode	PRM (parallel reaction monitoring)
spray voltage	3.5 kV
Auxiliary gas heater temperature	320°C
Capillary temperature	275°C
sheath gas flow rate	45
Auxiliary gas flow rate	10
Maximum injection time	50 ms

Table S8. PRM acquisition parameters for targeted metabolite quantification

Analyte	Abbreviation	Retention time (min)	Precursor ion (m/z)	Product ion (m/z)	Collision energy (eV)
Tryptophan	Trp	4.88	205.0975	188.0706; 146.060	10
5-Hydroxytryptophan	5-HTP	3.55	221.0921	204.0655; 162.0548	10
Serotonin	5-HT	2.88	177.1022	160.0756; 132.0807	15
Melatonin	MT	7.10	233.1284	174.0913; 216.1017	15
Kynurenine	Kyn	3.78	209.0921	192.0655; 94.0652	10
5-hydroxyindoleacetic acid	5-HIAA	5.45	192.0655	146.0601; 116.98598	15
[$^{15}N_2$]-L-tryptophan	L-Trp-$^{15}N_2$	4.88	207.0912	189.06749; 147.0570	10

Table S9. Characteristics of the calibration curves and estimated limits of detection (LOD) and quantification (LOQ) for the quantitative UHPLC–MS/MS method

Analyte	Sample type	Calibration range	Calibration equation	R^2	LOD	LOQ
Trp	Feces	2500-50000 ng/mL	$y = 3.322 \times 10^{-4}x - 6.243 \times 10^{-3}$	0.9978	596 ng/mL	1806 ng/mL
Trp	Plasma	2000-40000 ng/mL	$y = 3.5 \times 10^{-4}x - 7.954 \times 10^{-2}$	0.9983	283 ng/mL	857 ng/mL
Trp	Hippocampus	2000-24000ng/mL	$y = 3.912 \times 10^{-4}x - 2.406 \times 10^{-2}$	0.9990	337 ng/mL	1023 ng/mL
5-HTP	Feces	200-800 ng/mL	$y = 1.805 \times 10^{-4}x - 3.739 \times 10^{-5}$	0.9971	45.7 ng/mL	138.5 ng/mL
5-HTP	Plasma	15-250 ng/mL	$y = 1.973 \times 10^{-4}x - 2.070 \times 10^{-4}$	0.9907	3.35 ng/mL	10.14 ng/mL
5-HTP	Hippocampus	20-200 ng/mL	$y = 2.52 \times 10^{-4}x - 1.754 \times 10^{-5}$	0.9993	4.6 ng/mL	13.9 ng/mL
5-HT	Feces	300-2400 ng/mL	$y = 2.106 \times 10^{-4}x - 2.099 \times 10^{-3}$	0.9934	62.7 ng/mL	190 ng/mL
5-HT	Plasma	25-400 ng/mL	$y = 1.204 \times 10^{-4}x - 6.290 \times 10^{-6}$	0.9968	5.5 ng/mL	16.6 ng/mL
5-HT	Hippocampus	50-300 ng/mL	$y = 1.564 \times 10^{-4}x - 3.719 \times 10^{-5}$	0.9978	10.6 ng/mL	32.0 ng/mL
MT	Feces	3-72 pg/mL	$y = 3.716 \times 10^{-2}x + 3.732 \times 10^{-4}$	0.9977	0.71 pg/mL	2.15 pg/mL
MT	Plasma	2.5-150 pg/mL	$y = 3.310 \times 10^{-2}x + 6.814 \times 10^{-3}$	0.9979	0.60 pg/mL	1.81 pg/mL
MT	Hippocampus	1-10 pg/mL	$y = 3.468 \times 10^{-2}x + 7.975 \times 10^{-4}$	0.9995	0.24 pg/mL	0.72 pg/mL
Kyn	Feces	30-480 ng/mL	$y = 2.481 \times 10^{-4}x - 2.608 \times 10^{-3}$	0.9977	4.65 ng/mL	14.1 ng/mL
Kyn	Plasma	50-500 ng/mL	$y = 2.552 \times 10^{-4}X - 6.705 \times 10^{-3}$	0.9916	5.17 ng/mL	15.7 ng/mL

Analyte	Sample type	Calibration range	Calibration equation	R^2	LOD	LOQ
Kyn	Hippocampus	60-1000 ng/mL	$y = 3.280 \times 10^{-4}X - 1.876 \times 10^{-3}$	0.9935	12.1 ng/mL	36.6 ng/mL
5-HIAA	Feces	100-500 ng/mL	$y = 9.081 \times 10^{-5}x - 1.402 \times 10^{-4}$	0.9999	21.8 ng/mL	66.1 ng/mL
5-HIAA	Plasma	10-300 ng/mL	$y = 1.055 \times 10^{-4}X - 4.997 \times 10^{-4}$	0.9977	1.25 ng/mL	3.79 ng/mL
5-HIAA	Hippocampus	300-1200 ng/mL	$y = 2.179 \times 10^{-4}X - 1.973 \times 10^{-2}$	0.9982	45.4 ng/mL	137.7 ng/mL

Table S10. Accuracy and precision of QC samples during UHPLC–MS/MS analysis

Analyte	Sample type	Nominal QC concentration	Accuracy (%)	RSD (%)
Trp	Feces	40000 ng/mL	94.9	12.5
		25000 ng/mL	106.6	9.7
		7500 ng/mL	104.8	12.6
	Plasma	20000 ng/mL	89.2	13.2
		32000 ng/mL	90.7	9.4
		6000 ng/mL	87.6	13.2
	Hippocampus	20000 ng/mL	87.4	9.6
		12000 ng/mL	89.2	10.1

Analyte	Sample type	Nominal QC concentration	Accuracy (%)	RSD (%)
		5000 ng/mL	91.7	12.6
5-HTP	Feces	700 ng/mL	106.5	8.9
		500 ng/mL	105.7	10.6
		300 ng/mL	95.6	8.8
	Plasma	200 ng/mL	105.8	10.9
		120 ng/mL	92.4	10.2
		30 ng/mL	104.9	13.8
	Hippocampus	160 ng/mL	92.5	10.6
		100 ng/mL	88.8	11.7
		50 ng/mL	87.3	10.6
5-HT	Feces	2000 ng/mL	88.9	11.2
		1200 ng/mL	90.2	12.8
		500 ng/mL	86.4	10.3
	Plasma	320 ng/mL	94.4	12.2
		200 ng/mL	105.5	12.2

Analyte	Sample type	Nominal QC concentration	Accuracy (%)	RSD (%)
		50 ng/mL	106.8	13.0
	Hippocampus	250 ng/mL	106.9	9.2
		150 ng/mL	95.6	10.3
		75 ng/mL	101.7	12.9
MT	Feces	60 pg/mL	92.6	11.7
		30 pg/mL	104.5	10.8
		6 pg/mL	105.8	12.7
	Plasma	120 pg/mL	104.3	11.6
		60 pg/mL	93.4	10.5
		5 pg/mL	106.3	12.3
	Hippocampus	8 pg/mL	86.0	11.5
		5 pg/mL	91.9	12.9
		2 pg/mL	88.2	10.7
Kyn	Feces	240 ng/mL	88.7	11.2
		400 ng/mL	89.7	13.5

Analyte	Sample type	Nominal QC concentration	Accuracy (%)	RSD (%)
		80 ng/mL	91.3	13.4
	Plasma	400 ng/mL	94.7	8.9
		100 ng/mL	104.5	12.8
		200 ng/mL	92.2	10.2
	Hippocampus	120 ng/mL	102.1	12.8
		500 ng/mL	92.5	10.4
		800 ng/mL	103.2	11.9
5-HIAA	Feces	450 ng/mL	104.6	12.6
		300 ng/mL	90.7	11.2
		150 ng/mL	92.6	13.1
	Plasma	250 ng/mL	89.4	11.8
		150 ng/mL	93.3	13.5
		25 ng/mL	86.1	10.7
	Hippocampus	1050 ng/mL	106.6	12.1
		750 ng/mL	104.1	11.6

Analyte	Sample type	Nominal QC concentration	Accuracy (%)	RSD (%)
		450 ng/mL	94.8	13.4

QC samples were prepared by spiking known amounts of standards into corresponding biological matrices and analyzed in six replicates.

Table S11. Extraction recovery and matrix effects of target metabolites in different biological matrices by UHPLC–MS/MS analysis

Analyte	Sample type	Nominal QC concentration	Extraction recovery (%)	RSD (%)	Matrix effect (%)	RSD (%)
Trp	Feces	40000 ng/mL	82.2	10.7	108.9	12.7
		25000 ng/mL	80.8	7.1	113.7	12.8
		7500 ng/mL	78.5	9.5	110.1	12.7
	Plasma	20000 ng/mL	105.2	9.5	88.5	11.6
		32000 ng/mL	96.1	11.4	85.6	8.9
		6000 ng/mL	105.3	9.8	87.7	12.4
	Hippocampus	20000 ng/mL	90.3	10.5	86.1	11.6
		12000 ng/mL	82.1	8.0	82.7	10.7
		5000 ng/mL	80.9	8.1	85.2	8.0
5-HTP	Feces	700 ng/mL	105.4	6.5	106.6	10.1

Analyte	Sample type	Nominal QC concentration	Extraction recovery (%)	RSD (%)	Matrix effect (%)	RSD (%)
		500 ng/mL	101.7	10.1	107.6	9.5
		300 ng/mL	95.9	9.0	111.0	7.7
	Plasma	200 ng/mL	78.2	8.8	86.0	11.1
		120 ng/mL	83.3	10.9	90.0	8.2
		30 ng/mL	79.8	9.6	92.1	10.6
	Hippocampus	160 ng/mL	96.7	7.5	93.2	10.7
		100 ng/mL	104.1	7.3	87.0	9.9
		50 ng/mL	105.6	9.8	91.1	10.3
5-HT	Feces	2000 ng/mL	78.8	13.1	107.4	7.9
		1200 ng/mL	87.6	12.6	102.8	8.8
		500 ng/mL	80.9	11.3	97.5	10.1
	Plasma	320 ng/mL	87.3	8.8	89.6	10.9
		200 ng/mL	95.5	8.0	90.2	11.4
		50 ng/mL	88.7	11.2	86.3	10.1
	Hippocampus	250 ng/mL	79.4	7.1	107.8	9.0
		150 ng/mL	82.0	7.8	109.4	10.8

Analyte	Sample type	Nominal QC concentration	Extraction recovery (%)	RSD (%)	Matrix effect (%)	RSD (%)
		75 ng/mL	80.6	8.2	111.5	12.3
MT	Feces	60 pg/mL	82.1	12.7	97.7	11.9
		30 pg/mL	83.0	9.8	89.5	11.0
		6 pg/mL	90.2	13.6	91.3	10.8
	Plasma	120 pg/mL	77.8	9.3	86.5	12.2
		60 pg/mL	81.6	7.7	89.7	11.3
		5 pg/mL	84.0	10.8	85.8	11.6
	Hippocampus	8 pg/mL	95.4	9.9	108.3	9.7
		5 pg/mL	104.2	7.1	110.3	9.8
		2 pg/mL	103.9	8.3	102.9	7.7
Kyn	Feces	240 ng/mL	87.1	7.2	87.4	9.3
		400 ng/mL	79.4	11.5	85.2	8.2
		80 ng/mL	82.7	10.6	83.6	8.7
	Plasma	400 ng/mL	81.2	13.7	96.4	9.6
		100 ng/mL	78.3	11.0	98.0	7.0
		200 ng/mL	86.7	9.9	90.8	9.5

Analyte	Sample type	Nominal QC concentration	Extraction recovery (%)	RSD (%)	Matrix effect (%)	RSD (%)
	Hippocampus	120 ng/mL	86.0	12.2	87.2	7.6
		800 ng/mL	87.5	8.7	89.0	7.2
		500 ng/mL	84.2	11.9	85.1	12.2
5-HIAA	Feces	450 ng/mL	81.9	10.8	94.3	10.5
		300 ng/mL	81.8	8.2	85.5	12.2
		150 ng/mL	87.6	9.0	88.2	12.3
	Plasma	250 ng/mL	81.1	7.5	105.3	8.1
		150 ng/mL	83.6	8.5	109.0	11.5
		25 ng/mL	77.8	10.2	113.4	9.5
	Hippocampus	1050 ng/mL	83.1	10.7	86.4	8.0
		750 ng/mL	82.8	9.3	84.4	10.3
		450 ng/mL	77.9	10.6	92.7	10.8

Table S12. Sequencing depth and read-length statistics of 16S rRNA gene amplicons from individual fecal samples

	Sample_info	Seq_num	Base_num(bp)	Mean_length(bp)	Min_length(bp)	Max_length(bp)
B18	B18-1	70451	29481084	418.46225	216	431
	B18-2	76752	31991523	416.8168	232	540
	B18-3	67750	28178205	415.91447	318	458
	B18-4	82809	34513607	416.7857	258	443
	B18-5	66582	27756290	416.87378	337	484
CTR	CTR-1	65040	26993463	415.02864	251	535
	CTR-2	65369	27154564	415.40431	262	443
	CTR-3	68844	28531548	414.43769	216	433
	CTR-4	67840	28164480	415.16038	219	460
	CTR-5	68616	28519054	415.63271	317	443
DZP	DZP-1	71156	29660371	416.83584	339	509
	DZP-2	45416	18889505	415.92181	262	431
	DZP-3	59486	24915686	418.84958	240	444
	DZP-4	50338	21021345	417.6039	312	458
	DZP-5	65932	27781706	421.36908	337	443

Table S13. Composition of the modified MRS (mMRS) medium used for the cultivation of *Bifidobacterium longum* B18.

**Component**	**Final concentration**
Tryptone	10 g/L
Beef extract	10 g/L
Yeast extract	5 g/L
Glucose	20 g/L
Anhydrous sodium acetate	5 g/L
Dipotassium hydrogen phosphate trihydrate ($K_2HPO_4 \cdot 3H_2O$)	2.6 g/L
Diammonium hydrogen citrate	2 g/L
Magnesium sulfate heptahydrate ($MgSO_4 \cdot 7H_2O$)	0.1 g/L
Manganese sulfate monohydrate ($MnSO_4 \cdot H_2O$)	0.05 g/L
Tween 80	1 g/L
L-Cysteine	0.5 g/L
Agar	15 g/L

For liquid mMRS medium, the same formulation was used without agar.

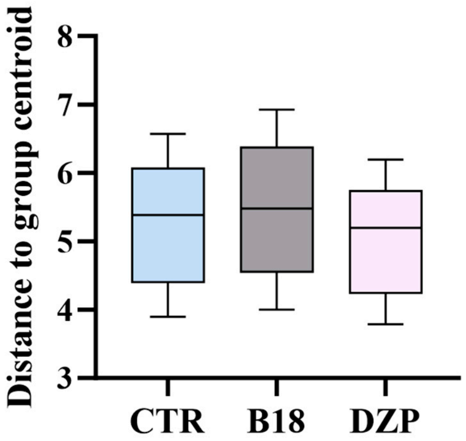

Figure S3. PERMDISP analysis of multivariate dispersion among the CTR, B18, and DZP groups. Distances of individual samples to their respective group centroids are shown as boxplots (n = 5 per group). Multivariate dispersion was assessed using PERMDISP based on binary Euclidean distances with 999 permutations. No significant difference in dispersion was detected among groups ($F = 2.840$, $P = 0.101$).

References

1. Rastogi, S.; Mittal, V.; Singh, A., In Vitro Evaluation of Probiotic Potential and Safety Assessment of *Lactobacillus mucosae* Strains Isolated from Donkey's Lactation. *Probiotics Antimicrob. Proteins* 2020, 12 (3), 1045-1056.
2. Li, B.; Ding, M.; Chen, C.; Zhao, J.; Shi, G.; Ross, P.; Stanton, C.; Chen, W.; Yang, B. *Bifidobacterium longum* subsp. infantis B6MNI Alleviates Collagen-Induced Arthritis in Rats via Regulating 5-HIAA and Pim-1/JAK/STAT3 Inflammation Pathways. *Journal of Agricultural and Food Chemistry.* 2023, 71, 17819–17832.
3. Dong, J.; Ping, L.; Cao, T.; Sun, L.; Liu, D.; Wang, S.; Huo, G.; Li, B. Immunomodulatory effects of the *Bifidobacterium longum* BL-10 on lipopolysaccharide-induced intestinal mucosal immune injury. *Frontiers in Immunology.* 2022, Volume 13–2022.
